# Early evolutionary history and genomic features of gene duplicates in the human genome

**DOI:** 10.1186/s12864-015-1827-3

**Published:** 2015-08-20

**Authors:** Lijing Bu, Vaishali Katju

**Affiliations:** Department of Biology, University of New Mexico, Albuquerque, NM 87131 USA; Department of Veterinary Integrative Biosciences, Texas A&M University, College Station, Texas, TX 77843-4458 USA

## Abstract

**Background:**

Human gene duplicates have been the focus of intense research since the development of array-based and targeted next-generation sequencing approaches in the last decade. These studies have primarily concentrated on determining the extant copy-number variation from a population-genomic perspective but lack a robust evolutionary framework to elucidate the early structural and genomic characteristics of gene duplicates at emergence and their subsequent evolution with increasing age.

**Results:**

We analyzed 184 gene duplicate pairs comprising small gene families in the draft human genome with 10 % or less synonymous sequence divergence. Human gene duplicates primarily originate from DNA-mediated events, taking up genomic residence as *intrachromosomal* copies in direct or inverse orientation. The distribution of paralogs on autosomes follows random expectations in contrast to their significant enrichment on the sex chromosomes. Furthermore, human gene duplicates exhibit a skewed gradient of distribution along the chromosomal length with significant clustering in pericentromeric regions. Surprisingly, despite the large average length of human genes, the majority of extant duplicates (83 %) are *complete* duplicates, wherein the entire ORF of the ancestral copy was duplicated. The preponderance of *complete* duplicates is in accord with an extremely large median duplication span of 36 kb, which enhances the probability of capturing ancestral ORFs in their entirety. With increasing evolutionary age, human paralogs exhibit declines in (i) the frequency of *intrachromosomal* paralogs, and (ii) the proportion of *complete* duplicates. These changes may reflect lower survival rates of certain classes of duplicates and/or the role of purifying selection. Duplications arising from RNA-mediated events comprise a small fraction (11.4 %) of all human paralogs and are more numerous in older evolutionary cohorts of duplicates.

**Conclusions:**

The degree of structural resemblance, genomic location and duplication span appear to influence the long-term maintenance of paralogs in the human genome. The median duplication span in the human genome far exceeds that in *C. elegans* and yeast and likely contributes to the high prevalence of *complete* duplicates relative to structurally heterogeneous duplicates (*partial* and *chimeric*). The relative roles of regulatory sequence versus exon-intron structure changes in the acquisition of novel function by human paralogs remains to be determined.

**Electronic supplementary material:**

The online version of this article (doi:10.1186/s12864-015-1827-3) contains supplementary material, which is available to authorized users.

## Background

The recent genomic era has established gene duplication as a dominant contributor to the origin of new genes and novel traits, which in turn fuels adaptation, niche diversification and increase in biocomplexity. Two characteristics of gene duplicates lend to their primacy in effecting evolutionary change, namely (i) their role in the creation of genetic redundancy or novel genes, and (ii) their high rate of spontaneous origin. The high supply rate of genetically and functionally redundant gene copies might be especially advantageous when the environment imposes immediate selection for increased gene dosage and gene expression [1]. The promiscuity of the gene duplication process leading to the duplication of DNA segments across gene boundaries, often in conjunction with the inclusion of noncoding DNA sequence to yield a novel open reading frame, can additionally yield new genes with distinctly novel functions [2, 3]. Notable examples of the fashioning of novel genes from the incomplete duplication of ancestral gene sequences account for the origin of antifreeze glycoproteins in Antarctic fish [4, 5] and the evolution of hermaphroditism in *Caeonorhabditis elegans* from an obligately outcrossing ancestor [6]. The second salient characteristic of gene duplicates is their astoundingly high rates of spontaneous origin. Empirical estimates of locus-specific or genome-wide spontaneous rates of gene duplication range from 10^−3^ to 10^−7^ per gene per generation [7, 8]. These high rates of gene duplication directly contribute to the high frequency of copy-number variants (CNVs) being uncovered in population-genomic studies [9–11].

Classical models of gene duplication make the key assumption that duplicated genes originate structurally and functionally redundant to the ancestral copy. An evolutionary trajectory leading to the origin of a hitherto novel function is thought to occur under a regime of relaxed selective constraints due to gradual accumulation of previously ‘forbidden’ deleterious mutations [12]. However, unbiased studies of entire age-cohorts of evolutionarily young gene duplicates in a few species have demonstrated the existence of gene copies bearing structural heterogeneity (*partial* or *chimeric* gene duplicates) due to incomplete duplication across ORFs and/or recruitment of novel noncoding sequences [13–16]. With respect to small segmental duplication (SSD) events, the frequency of *complete* gene duplicates (entire duplication of an ancestral ORF) can be highly variable; 39 % in *C. elegans* [13], 41-44 % in *Drosophila* species [14, 16] and 89 % in *Saccharomyces cerevisiae* [15]. Additionally, gene duplication via retrotransposition, which results in the insertion of the duplicate copy in a random location in the genome, likely engenders acquisition of novel regulatory elements and altered gene expression patterns. These *heterogeneous* gene duplicates (*partial*, *chimeric*, and *retrotransposed*) are more likely to be nonfunctionalized but also have the potential to gain immediate novel functions [3]. The diverse structural classes of gene duplicates, if identified in their early evolutionary existence, can provide insights into the mutational mechanisms underlying their origin as well as the sequence alterations that facilitate molecular innovations [3]. To date, we have a limited understanding of the population dynamics and selective constraints influencing different structural classes of gene duplicates. A comparative study of gene duplicates with low synonymous divergence in the *C. elegans* and *S. cerevisiae* genomes implied that both species–specific differences in mutational input and strength of natural selection moulded the distribution of gene duplicates in these two genomes [15].

Investigating the interplay between evolutionary forces and mutation in patterning the distribution of gene duplicates in the human genome might be of particular interest for several reasons. First, there has been a spate of population-genomic studies establishing widespread copy-number variation in humans and other hominoid and primate species [9, 17–19]. Second, segmental gene duplications have demonstrated a signature of expansion in early hominoid evolution [20]. Whereas a large fraction of the chromosomal rearrangements created by segmental duplications in humans are implicated in Mendelian and complex genetic disease [21–24], they additionally serve as important substrates for the origin of evolutionary innovations. Although the most common fate of gene duplicates may be immediate pseudogenization upon arrival, the extraordinary high rates of spontaneous gene duplication likely have a substantial influence on the trajectory of evolution by enabling the origin of discernible numbers of gene substrates for neofunctionalization [8]. In the context of human evolution, there is substantial interest in delineating the genetic changes that account for the emergence of human-specific morphological and behavioural changes since their divergence from other primates. Given the role of gene duplication in the emergence of evolutionary novelties and their high spontaneous rates of origin, human-specific gene duplicates would appear to be a promising avenue for investigation. Two notable examples of adaptive copy-number changes in humans involve the *AMY1* [25] and *SRGAP2C* [26, 27] genes.

To date, there has been no systematic study in a strict evolutionary context that comprehensively characterizes the structural and genomic features of a large, unbiased population of evolutionarily young gene duplicates in the human genome. Such a study would provide a rich natural history perspective on the mutational origins of human gene duplicates, the degree of structural resemblance between paralogs, and the patterns of genomic traffic in the early stages of their evolution. In addition, it would enable future comparative genomic research investigating differences in the genomic architecture of human- and chimpanzee-specific gene duplicates. Structural and genomic features of novel paralogs at inception can greatly influence their evolution and ultimate fate. In order to test the importance of structural features on the evolution of young gene duplicates, we performed a genome-wide survey of the entire population of evolutionarily young paralogs belonging to small gene-families in the human genome. Because subsequent mutational events in the evolutionary life of gene duplicates can rapidly erode their key characteristics at inception, we limited our analyses to putative evolutionarily young gene duplicates (synonymous divergence per synonymous site *K*_*S*_ ≤ 0.10) in the current human genome assembly with the similarity search cutoff capable of capturing paralogs with differing levels of structural resemblance. To our knowledge, this study is the first to delineate the relative fractions of *complete*, *partial*, and *chimeric* paralogs within an unbiased population of gene duplicates in the human genome.

## Results

We identified 184 human gene duplicate pairs belonging to small gene families (≤5 members) with low synonymous sequence divergence of 10 % or less (*K*_*S*_ ≤ 0.1) (Additional file 1: Table S1). Because the evolutionary dynamics of paralogs in large multigene families may differ markedly from those of paralogs comprising small gene families, we restricted our analyses to human paralogs belonging to families comprising five or less paralogs. The chromosomal location was confirmed for both paralogs belonging to 172 pairs. The remaining 12 pairs comprised at least one paralog located on a supercontig with an unassigned chromosomal location. Additional file 1: Table S1 lists the identification numbers of all paralogs comprising the 184 human gene duplicate pairs in conjunction with other relevant information such as synonymous divergence between paralogs, chromosomal location of the two paralogs, the assigned category of structural resemblance, transcriptional orientation of paralogs, duplication span (bp) and physical distance between paralogs located on the same chromosome.

### Assessment and controlling for the role of ectopic gene conversion in confounding evolutionary age estimates of paralogous sequences

We tested all 184 duplication events in our study for signatures of gene conversion using a chimpanzee ortholog as an outgroup sequence. We found evidence for gene conversion in the coding sequences of 26 of the 184 duplicate pairs tested, comprising 18 single-locus duplications and eight linked sets representing the duplication of more than one protein-coding ORF during a single duplication event. We conducted all subsequent statistical analyses of the genomic and structural features of human paralogs on two separate data sets: (i) all 184 duplicate pairs including the 26 sets that exhibited a positive signature of gene conversion, and (ii) 158 duplicate pairs by excluding 26 sets showing evidence of gene conversion. The exclusion of the 26 duplicate sets showing evidence of gene conversion did not qualitatively alter our results. For each subsequent analyses that involves *K*_*S*_ as a parameter, we report the significance values of statistical tests with and without inclusion of the 26 duplicate sets exhibiting evidence of gene conversion. Furthermore, we conducted all analyses by alternatively including and excluding paralogs residing on the sex chromosomes. The exclusion of sex-linked paralogs did not change our conclusions (Additional file 2: Figures S1-S6).

### L-shaped frequency distribution of human gene duplicates

**Fig. 1:**
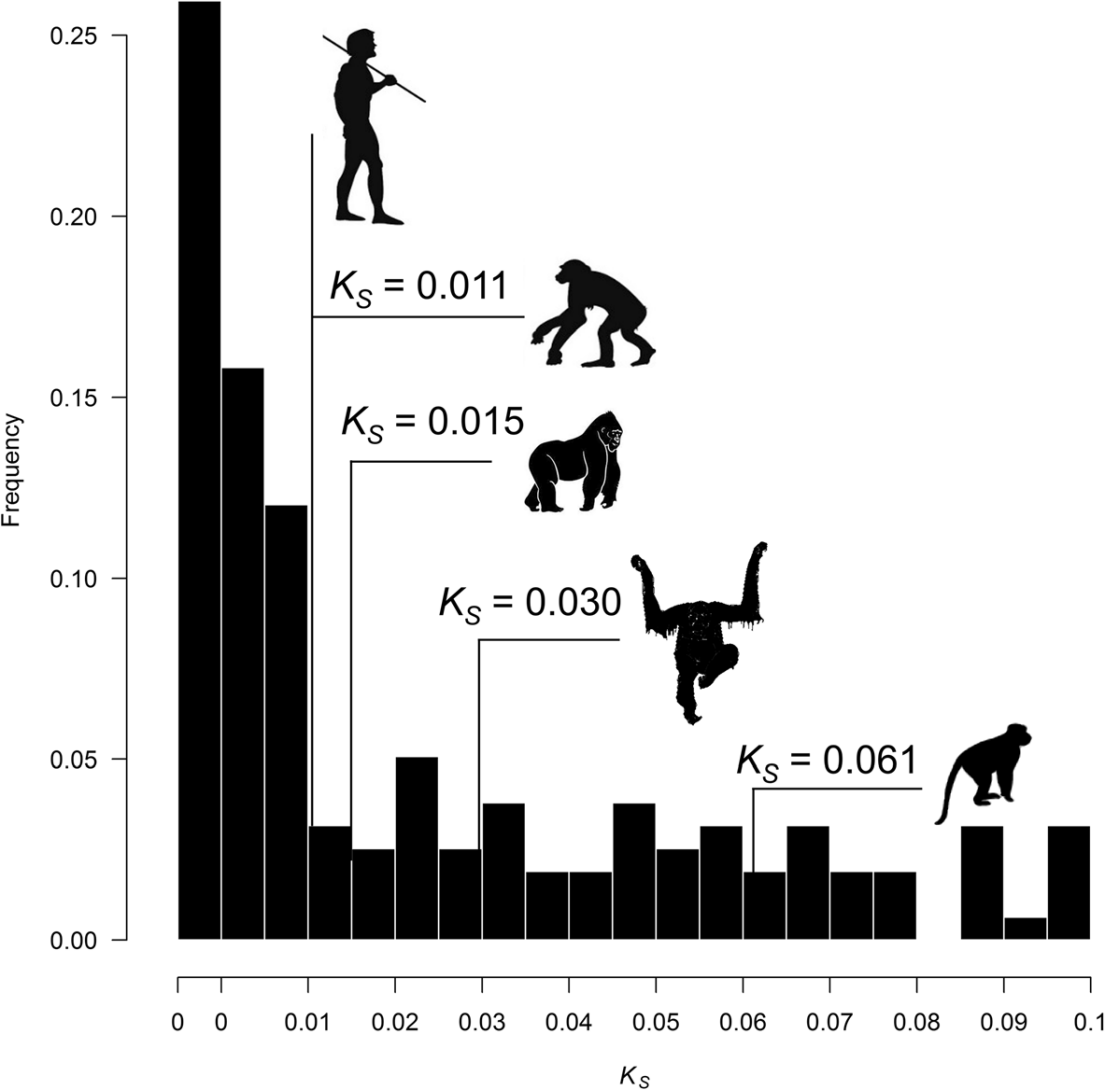
Synonymous changes per synonymous site (*K*_*S*_) based age distribution of 184 human gene duplicate pairs. The average *K*_*S*_ between coding regions of human versus chimpanzee, gorilla, orang-utan, and macaque [28, 67] is shown for scale, and suggests that a large fraction of human gene duplicates within this data set may have originated since the human-chimpanzee split

Assuming that the synonymous sequence divergence between paralogs is an adequate proxy for evolutionary time, the *K*_*S*_ values between paralogs were used to generate a relative age-distribution of the focal 184 human gene duplicate pairs (Fig. 1). The distribution of putative evolutionarily young human gene duplicates is strongly L-shaped with the highest density of gene duplicates occurring in the youngest age cohorts and a strong decline in gene duplicate frequencies with increasing synonymous divergence. The youngest age-cohort of human gene duplicates (*K*_*S*_ = 0), which we refer to as the ‘newborn’ cohort, notably comprises more than 40 % of all duplicate pairs within our data set. Moreover, >50 % of the young gene duplicates identified in humans have lesser divergence at synonymous sites than the average divergence between human and chimpanzee orthologs (*K*_*S*_ = 0.011) [28]. We found that 103 of 184 young duplicate pairs (56 %) in humans correspond to young gene duplicates in the chimpanzee genome and therefore may have occurred before the human-chimpanzee split. Some smaller proportions of human gene duplicates within this study are also expected to be shared with more distantly-related Great Ape species and other old-world primate species such as rhesus macaques. The exclusion of 26 duplicate sets showing evidence of gene conversion did not alter the overall L-shaped frequency distribution of human gene duplicates, with a significant portion of the evolutionarily recent gene duplicates occurring since the human-chimpanzee split.

### Genome distance between human paralogs as a function of evolutionary age

Where do newborn gene duplicates take up residence in the genome and does the pattern of distribution change with increasing evolutionary age? We used two measures to infer the genomic distribution of paralogs in the human genome, namely (i) the chromosomal location (*intra-* vs. *interchromosomal* locations for paralogs residing on the same and different chromosomes, respectively) and (ii) the genomic distance (unique sequence in bp) separating two *intrachromosomal* paralogs as a function of synonymous divergence, *K*_*S*_. These two analyses were restricted to 172 gene duplicate pairs with known chromosomal locations for both paralogs.

**Fig. 2:**
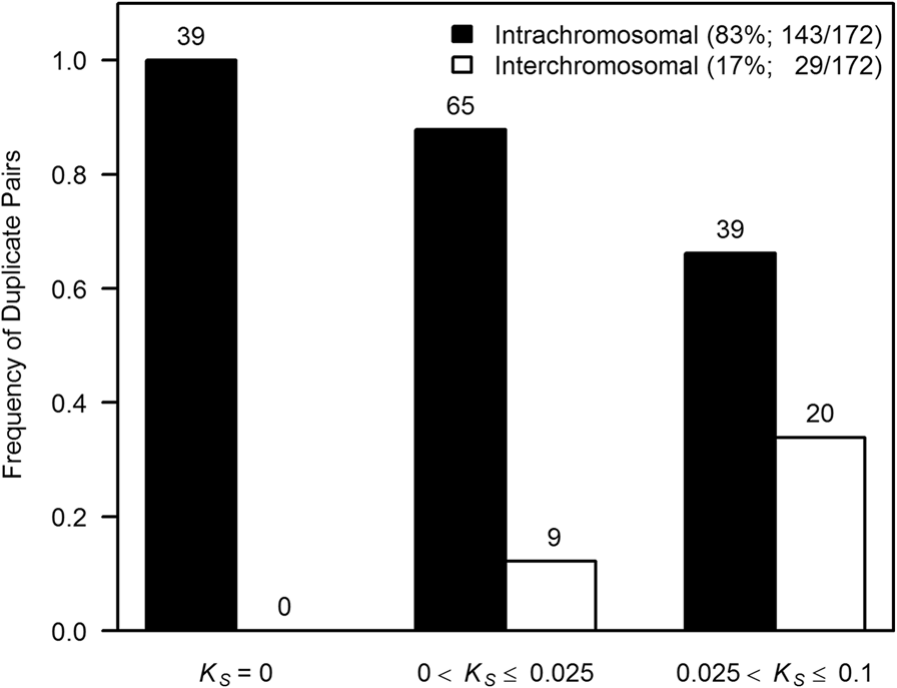
Composition frequencies of *intra*- and *interchromosomal* duplication within three age-cohorts of human gene duplicate pairs. The sample sizes of duplicate pairs within each age category (*K*_*S*_ = 0, 0 < *K*_*S*_ ≤ 0.025, and 0.025 < *K*_*S*_ ≤ 0.1) are provided above the corresponding bars. The total sample size comprised 172 duplicate pairs with assigned chromosomal locations for both paralogs. Chromosomal location is significantly associated with the *K*_*S*_ values for paralogs (*G* = 25.1, *p* = 3.59 × 10^−6^)

With respect to chromosomal location, 83 % (143/172) of the entire data set of 172 gene duplicate pairs comprise *intrachromosomal* duplications with both paralogs residing on the same chromosome; the remaining 17 % (29/172) pairs display *interchromosomal* location of the two paralogs (Fig. 2). The exclusion of 26 duplicate pairs exhibiting gene conversion resulted in 82 % (121/148) *intrachromosomal* and 18 % (27/148) *interchromosomal* duplications, respectively. We further investigated whether the relative frequencies of *intrachromosomal vs. interchromosomal* duplicates was altered with increasing evolutionary age by classifying the human duplicate pairs into three evolutionary age-cohorts (*K*_*S*_ = 0, 0 < *K*_*S*_ ≤ 0.025, and 0.025 < *K*_*S*_ ≤ 0.1). Although *intrachromosomal* duplicates dominate in frequency within each of the three age-cohorts, a clear decline in the frequency of *intrachromosomal* duplicates (and increase in the frequency of *interchromosomal* duplicates) is apparent as a function of increasing synonymous divergence: 100 (39/39), 88 (65/74), and 66 % (39/59) from evolutionarily younger to older age-cohorts (Fig. 2). A *G*-test of independence revealed chromosomal location to be significantly associated with synonymous divergence between paralogs (*G* = 25.1, *df* = 2, *p* = 3.59 × 10^−6^). This significant trend of frequency decline of *intrachromosomal* duplicates with increasing evolutionary age remains unaltered even when the 26 duplicates pairs with signatures of gene conversion are excluded from the analyses (*G* = 23.2, *df* = 2, *p* = 9.35× 10^−6^). RNA-mediated gene duplicates appear to be older on average (higher *K*_*S*_) and more likely to be found on different chromosomes. These biases in the features of RNA-mediated duplications may be responsible for the apparent relationship between chromosomal location (*intra-* vs. *interchromosomal*) and evolutionary age (*K*_*S*_). However, when 21 putative RNA-mediated gene duplicate pairs were excluded from the analysis, we still found a significant increase in the proportion of *interchromosomal* duplicates with evolutionary age (*G* =10.2, *df* = 2, *p* = 0.006).

**Fig. 3:**
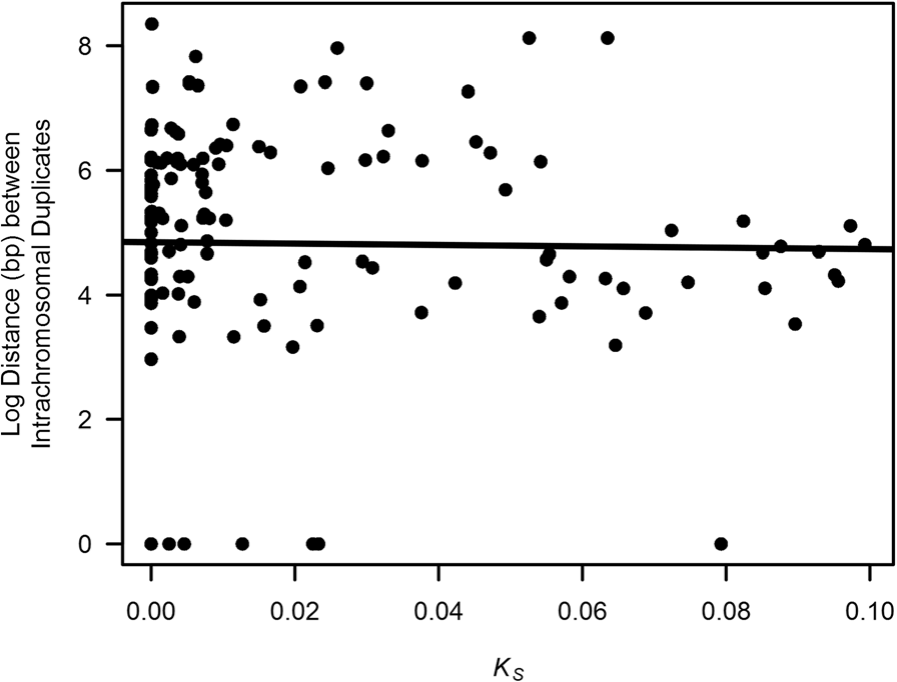
The physical distance between *intrachromosomal* gene duplicates as a function of *K*_*S*_. The regression line represents the relationship between distance separating all *intrachromosomal* paralogs (143 pairs with *K*_*S*_ ≤ 0.1) and *K*_*S*_. The correlation between *K*_*S*_ and distance between paralogs is not significant (*r* = −0.08, *df* = 141, *p* = 0.84)

When only *intrachromosomal* paralogs within our data set of duplicate pairs with *K*_*S*_ ≤ 0.1 were analyzed (143 duplicate pairs), the correlation between *K*_*S*_ and log (distance) is not significant (*r* = −0.08, *df* = 141, *p* = 0.84) (Fig. 3), suggesting no increase in genomic distance between *intrachromosomal* paralogs over evolutionary time. The results were qualitatively the same when 22 *intrachromosomal* duplicate sets with a signature of gene conversion were omitted from the analysis (*r* = −0.09, *df* = 119, *p* = 0.87).

### Chromosomal distribution of gene duplicates

**Fig. 4:**
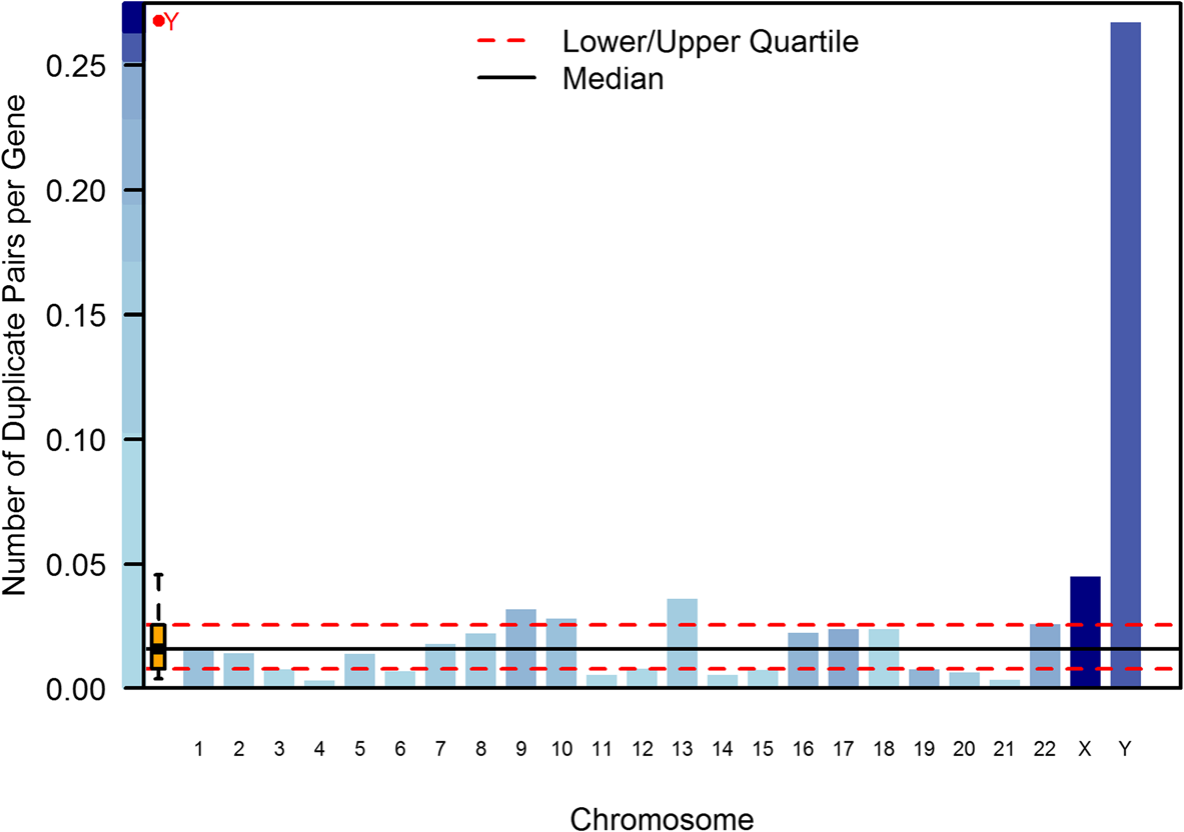
Nonrandom chromosomal distribution of 172 pairs of young gene duplicates in the human genome. The height of the blue bars indicates the relative duplication frequencies across 24 chromosomes, calculated as the ratio of the number of duplicate copies on a chromosome and the number of protein-coding genes on the same chromosome. The box plot displays the variation in these relative frequencies across 24 chromosomes, with the median represented by a solid line and the upper and lower quartiles in dotted lines. There was a significant difference in the frequency of duplicates between chromosomes (*G* = 36.8, *p* = 0.034) but no significant difference among the autosomes (*G* = 21.9, *p* = 0.405)

Are gene duplicates randomly distributed across all 24 chromosomes in the human genome or are they clustered on certain chromosomes? To correct for the variable number of protein-coding genes among chromosomes, we normalized the data by plotting the number of duplicate pairs/number of protein-coding genes per chromosome. Duplicated genes appear to be more frequent on the sex chromosomes than on the autosomes, but randomly distributed among autosomes. A *G*-test of differences in the frequency of *intrachromosomal* duplications among chromosomes was significant (*G* = 37.53, *df* = 23, *p* = 0.029), but not significant when only autosomes were considered (*G* = 24.52, *df* = 21, *p* = 0.27). When all duplicates (*intra-* and *interchromosomal*) in our study were considered, there was a significant difference in the frequency of duplications across chromosomes (*G* = 36.8, *df* = 23, *p* = 0.034) (Fig. 4), but no significant difference when only autosomes were considered (*G* = 21.9, *df* = 21, *p* = 0.405). Chromosomes X and Y have approximately three- and 17-fold more duplicates, respectively, than expected under an assumption of equal duplication frequencies across all chromosomes. The exclusion of 26 duplicate sets with evidence of gene conversion did not qualitatively change the above results (*intrachromosomal* duplications across all chromosomes: *G* = 43.99, *df* = 23, *p* = 0.0052; *intrachromosomal* duplications across all autosomes: *G* = 28.73, *df* = 21, *p* = 0.1206; *intra-* and *interchromosomal* duplications across all chromosomes: *G* = 42.07, *df* = 23, *p* = 0.0089; *intra-* and *interchromosomal* duplications across all autosomes: *G* = 25.3, *df* = 21, *p* = 0.234).

**Fig. 5:**
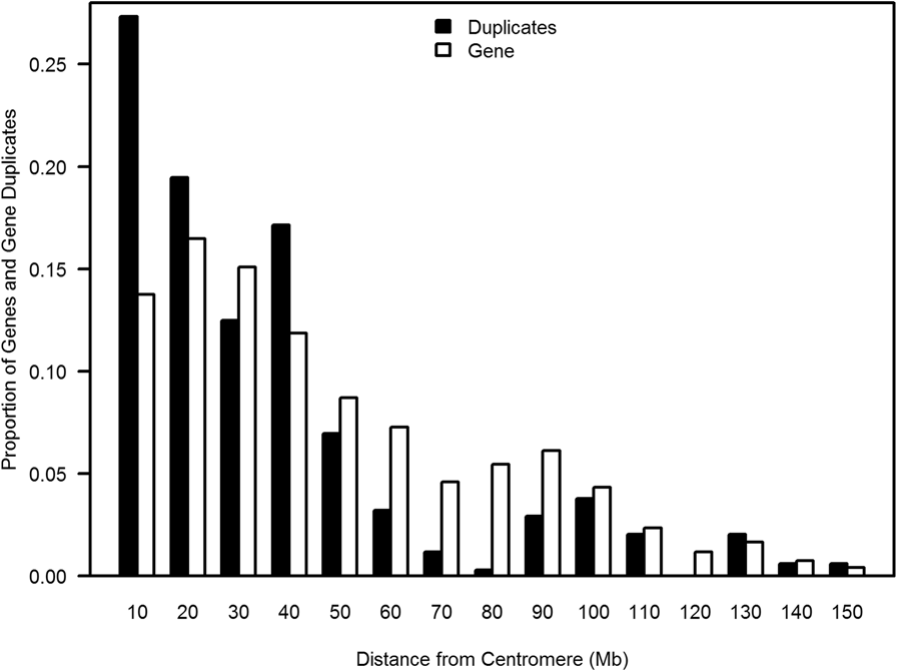
Location of 172 human gene duplicates relative to the centromere. The relative location of gene duplicates along chromosomal arms deviates significantly from an expected distribution based on protein-coding gene enrichment. Each chromosome was subdivided into 10 Mb bins representing increasing distance from the centromere. The proportions of gene duplicates and protein-coding genes (*N* = 20,172) within each bin are represented by black and white bars, respectively. The distribution of gene duplicates along the chromosomes deviates significantly from a random expectation (*G* = 54.9, *p* = 8.96 × 10^−7^)

We further investigated if the distribution of human gene duplicates occurs in a random fashion along the length of a chromosome or exhibits a biased gradient of location, in proximity to the centromeres. The distribution of gene duplicates along the length of chromosomes shows significant deviation from a random expectation based on gene density on chromosomes (*G* = 54.9, *df* = 14, *p* = 8.96 × 10^−7^). Collectively, regions within 10 Mb distance from the centromeres appear to be particularly enriched for gene duplicates (Fig. 5). The exclusion of 26 duplicate sets with evidence of gene conversion did not qualitatively change the above results (*G* = 54.18, *df* = 14, *p* = 1.2 × 10^−6^).

### Equal proportions of *intrachromosomal* paralogs with direct and inverse transcriptional orientation

Does the orientation of a duplicated gene relative to its ancestral gene influence its chances of survival? Of 143 young gene duplicates on the same chromosome, there are 46 (66/143) and 54 % (77/143) duplicates with *direct* and *inverse* transcriptional orientation, respectively. However, the proportion of inverted duplications is not significantly greater than those with the same (*direct*) transcriptional orientation (*G* = 0.844, *df* = 1, *p* = 0.36). The exclusion of 22 *intrachromosomal* duplicate sets with evidence of gene conversion did not qualitatively change the above results, finding no significant difference in the proportion of *direct* (45 %; 54/121) versus *inverted* (55 %; 67/121) duplicates (*G* = 1.39, *df* = 1, *p* = 0.24). A comparison of three age-cohorts of gene duplicates (*K*_*S*_ = 0, 0 < *K*_*S*_ ≤ 0.025, and 0.025 < *K*_*S*_ ≤ 0.1) detected no difference in the relative proportions of *direct vs. inverse* duplicates (*G* = 1.7949, *df* = 2, *p* = 0.41), suggesting no change in their frequencies with increasing evolutionary age. An identical trend was observed when 22 *intrachromosomal* duplicate sets with gene conversion were excluded from the analyses (*G* = 1.63, *df* = 2, *p* = 0.44).

### Predominance of young gene duplicates with complete structural resemblance in the human genome

**Fig. 6:**
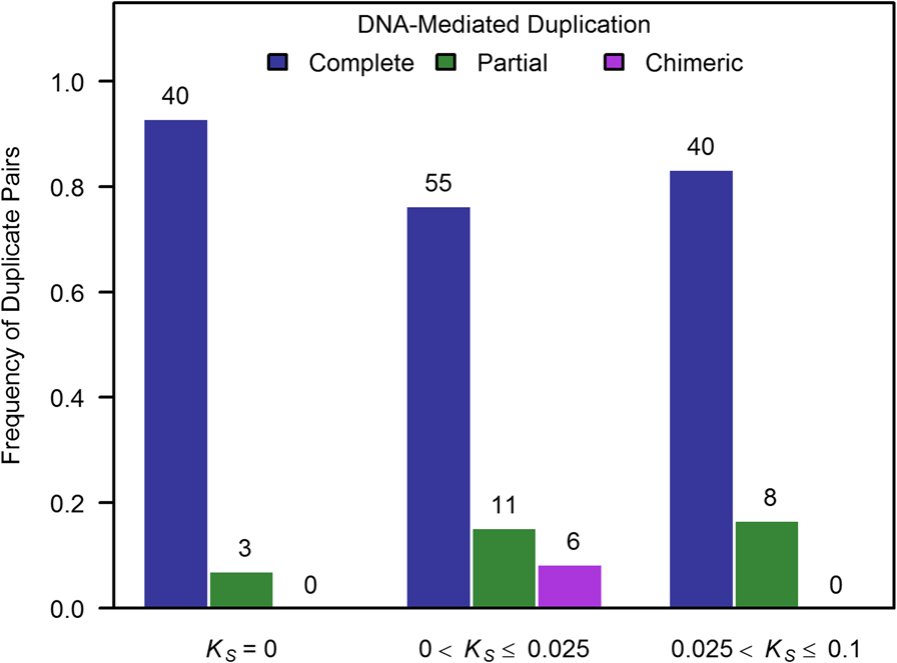
Composition frequencies of three structural categories of DNA-mediated gene duplicates across three evolutionary age-cohorts. The sample sizes of duplicate pairs within each of the three categories (*K*_*S*_ = 0, 0 < *K*_*S*_ ≤ 0.025, and 0.025 < *K*_*S*_ ≤ 0.1) are provided above the corresponding bars (*N* = 163 gene duplicate pairs). There was a significant difference in the relative proportions of the three structural categories of gene duplicates (*G* = 11.9, *p* = 0.018) as a function of evolutionary age, *K*_*S*_

The structural resemblance between gene paralogs can influence their evolutionary dynamics. For DNA-mediated duplication events (*N* = 163 duplicate pairs), paralogs bearing *complete* structural resemblance dominate the sample of young human gene duplicates. The frequencies of *complete*, *partial*, and *chimeric* gene duplicates within our data set were 83, 13, and 4 %, respectively. *Complete* duplicates represent the most common structural category even when gene duplicates of varying evolutionary age were analyzed (cohorts *K*_*S*_ = 0, 0 < *K*_*S*_ ≤ 0.025, and 0.025 < *K*_*S*_ ≤ 0.1). However, the proportion of *complete* duplicates declines with evolutionary age (Fig. 6), comprising 93, 76, and 83 % of the total duplicate pairs in the *K*_*S*_ = 0, 0 < *K*_*S*_ ≤ 0.025, and 0.025 < *K*_*S*_ ≤ 0.1 age-cohorts, respectively. Furthermore, there was a significant difference in the relative proportions of the three structural categories of gene duplicates (*G* = 11.9, *df* = 4, *p* = 0.018) as a function of evolutionary age as represented by three different age-cohorts of gene duplicates (*K*_*S*_ = 0, 0 < *K*_*S*_ ≤ 0.025, and 0.025 < *K*_*S*_ ≤ 0.1). This significant difference in the relative proportions of the three structural categories of gene duplicates as a function of *K*_*S*_ was also observed when 26 duplicate sets with gene conversion were excluded from the analyses (*G* = 11.87, *df* = 4, *p* = 0.018).

### Duplication span exceeds the average gene length in the human genome

**Fig. 7:**
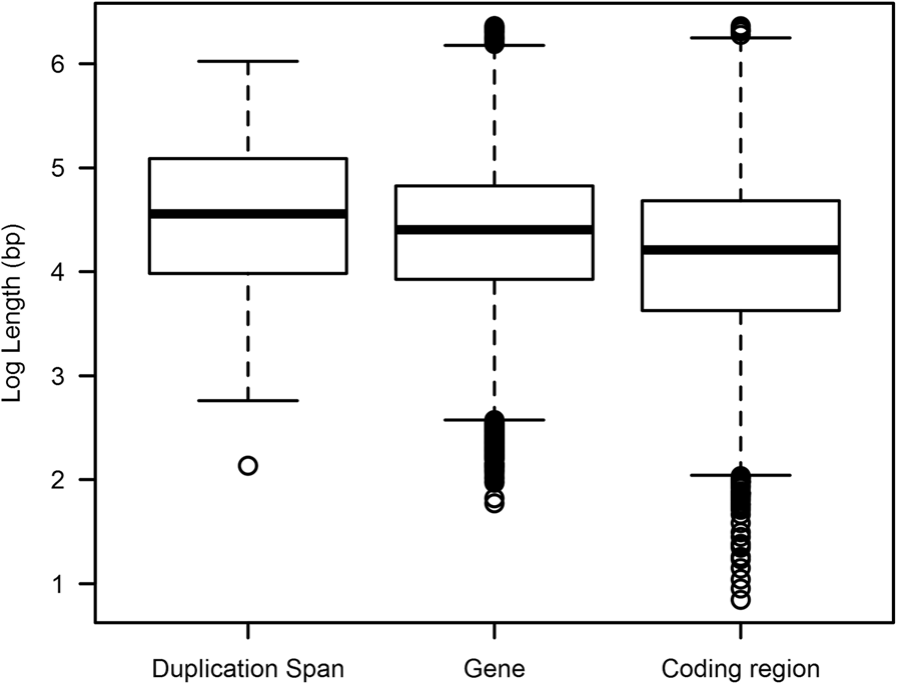
Box plot displaying the distribution of minimum duplication span for 184 human young gene duplicates. The range and median length of human protein-coding genes and their coding regions are displayed for comparison. The median duplication span of human paralogs is significantly greater than the median gene length in the human genome (*W* = 2,102,894, *p* = 0.0015) as well as the median length of the coding region for protein-coding genes (*W* = 2,367,542, *p* = 7.6 × 10^−11^)

**Fig. 8:**
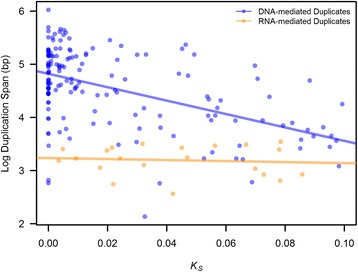
Duplication span of DNA- and RNA- mediated duplicates as a function of evolutionary age (*K*_*S*_). The data set comprises 163 DNA-mediated duplicate pairs (blue) and 21 RNA-mediated duplicate pairs (orange). The span of DNA-mediated duplications shows a significant decrease with evolutionary age (Kendall’s Tau = −0.258, *p* = 2 × 10^−6^)

The length of the duplication tract, which we refer to as the *duplication span*, is an important characteristic of gene duplicates that has bearing on the structural features of newly duplicated genes as well as aspects relating to gene dosage. For example, short or abbreviated duplication spans are less likely to duplicate an ancestral ORF in its entirety. Very lengthy duplication spans are more likely to duplicate multiple ORFs and increase the probability of detrimental changes relating to gene dosage. What is the length distribution of duplication tracts involving protein-coding sequences in the human genome? The coding regions (from the initiation codon to the termination codon) of human protein-coding genes have a median and mean length of 25 and 65 kb, respectively. The duplication span within our data set of human gene duplicate pairs ranged from 136 bp - 1,055 kb, with a median and mean value of 36 and 86 kb, respectively. The duplication span of young human gene duplicates is significantly greater than the human gene length (Wilcoxon Rank Sum Test, *W* = 2,102,894, *p* = 0.0015) as well as the length of the coding region for protein-coding genes (Wilcoxon Rank Sum Test, *W* = 2,367,542, *p* = 7.61 × 10^−11^) (Fig. 7). The span of DNA-mediated duplications shows a significant decrease with evolutionary age (Kendall’s Tau = −0.258, *p* = 2 × 10^−6^) (Fig. 8). This significant reduction in the span of paralogs formed by DNA-mediated duplication events is observed even when 26 duplicate sets with gene conversion were excluded from the analyses (Kendall’s Tau = −0.242, *p* = 4.4 × 10^−5^). In contrast, there is no significant change in the span of putative retrotransposed duplicates as a function of *K*_*S*_ (entire data set, Kendall’s Tau = 0, *p* = 1; exclusion of 26 duplicate sets with evidence of gene conversion, Kendall’s Tau = −0.041, *p* = 0.83) (Fig. 8).

### Smaller, but persistent presence of RNA-mediated duplications in human evolution

**Fig. 9:**
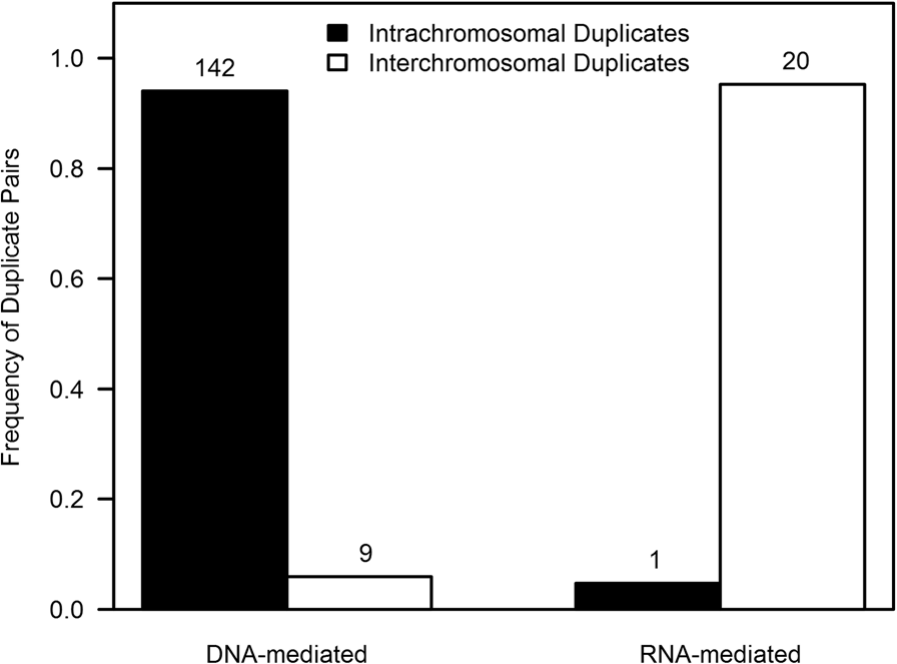
Composition frequencies of *intra-* versus *interchromosomal* gene duplicates within DNA-mediated and RNA-mediated duplication events. The genomic distribution of *retrotransposed* gene duplicates is significantly different from their DNA-mediated counterparts (*G* = 76.04, *p* = 2.2 × 10^−6^)

What is the frequency and fate of RNA-mediated duplication events relative to DNA-mediated ones in the human genome? Within our data set of 184 human duplicate pairs, 11.4 % (21/184) were identified as putative *retrotransposed* gene duplicates. Interestingly, putative *retrotransposed* gene duplicates were completely absent in the youngest *K*_*S*_ = 0 age-cohort although their proportions appear to increase with age; 10 and 21 % of all gene duplicates in the 0 < *K*_*S*_ ≤ 0.025 and 0.025 < *K*_*S*_ ≤ 0.1 age-cohorts, respectively. Furthermore, the genomic distribution of *retrotransposed* gene duplicates is significantly different from their DNA-mediated counterparts (*G* = 76.04, *df* = 1, *p* = 2.2 × 10^−6^). As expected, *retrotransposed* gene duplicates are predominantly *interchromosomal* whereas the majority of DNA-mediated duplication events yield *intrachromosomal* paralogs (Fig. 9). Of the 21 *retrotransposed* gene duplicates, seven and zero duplicate pairs had one paralog located on the X and Y chromosome, respectively. With respect to the seven *retrotransposed* duplicate pairs with one paralog residing on the X chromosome, four paralogs had intact introns and three paralogs were lacking introns, thereby suggesting approximately equal rates of traffic from and to the X chromosome.

## Discussion

Structural and genomic features of recent gene duplicates can have important consequences for their evolutionary fate. For instance, gene duplications that contain the complete coding and regulatory sequences of the ancestral gene are more likely to have conserved the ancestral function compared to gene duplications that are incompletely duplicated. Similarly, gene duplicates that alter their genomic location or transcriptional orientation are more likely to be expressed differently from their ancestral paralogs. While human paralogs have been intensively studied in the last decade as a class of mutations within population-genomic studies investigating copy-number variants, a systematic and unbiased investigation delineating their basic structural and genomic features at, or close to inception, has been lacking.

We compared the various genomic and structural features of human paralogs belonging to different age-cohorts to determine if any patterns are altered with increasing evolutionary age. It is possible that some very recent gene duplications have been missed. However, these potential omissions should not influence our conclusions unless the chromosomal distribution, structural categories and orientation of recent undetected gene paralogs are different from the known gene duplications. We applied the same methodology to conduct our analyses of human gene duplicates as used previously for *C. elegans* and yeast paralogs [13, 15] to facilitate direct comparison of the spectrum and properties of paralogs across these diverse eukaryotic genomes.

Ectopic gene conversion between homologous sequences, a form of concerted evolution, can homogenize the sequences of evolutionary older paralogs and lead to erroneous estimates of their evolutionary age as measured by the degree of synonymous divergence between paralogs (*K*_*S*_). Although we currently lack any genome-wide direct empirical estimates of the spontaneous rate of ectopic gene conversion in humans or other species, it appears to be a ubiquitous process leading to sequence homogenization between paralogs in virtually all organisms that have been studied including humans [29–41]. A high rate of ectopic gene conversion between members of duplicates pairs could contribute, in some part, to the higher frequencies of gene duplicates in the younger age-cohorts and thereby influence conclusions regards their evolutionary dynamics. While several studies have demonstrated evidence for frequent gene conversion among human paralogs [40, 41], a study of four mammalian genomes including humans found a minimal contribution of ectopic gene conversion in the evolution of young gene duplicates [42]. Furthermore, Semple and Wolfe [35] demonstrated that the frequency of ectopic gene conversion events in *C. elegans* is positively correlated with gene-family size. To guard against the confounding effects of gene conversion in our understanding of the early evolutionary dynamics of human paralogs, we restricted our data set to putatively young paralogs in small gene-families of five members or less. The inclusion or exclusion of the 26 duplicate pairs with a signature of gene conversion did not qualitatively alter our results pertaining to the evolutionary dynamics of human paralogs.

In concordance with genome-wide studies of extant gene duplicates in humans and other species [43], the distribution of human gene duplicates with low synonymous sequence divergence is strongly L-shaped, with 23 % of the paralogs being identical at synonymous sites. The highest density of gene duplicates occurs in the youngest (*K*_*S*_ = 0) age-cohort followed by a strong decline in gene duplicate frequencies with increasing synonymous divergence. Although positive selection has been implicated in the spread and maintenance of some human gene duplicates, the most obvious explanation for this trend of continuing decline of duplicates with increasing synonymous divergence is a high rate of duplicate gene loss and suggests that a large fraction of the recent gene duplicates still lingering in our genomes are either evolving neutrally under drift conditions, or being exposed to weak negative selection [44].

The degree of structural resemblance between paralogs has implications for the evolution of functionally novel genes following duplication. It has been argued that the evolution of novel functions in a new gene duplicate may be facilitated by radical changes in the exon-intron structure of the derived copy, typically manifest in structurally heterogeneous paralogs comprising *partial* and *chimeric* duplicates [3]. As such, *partial* and *chimeric* duplicates may be worthy candidate genes for investigations into the genetic basis of human-specific traits. Indeed, certain novel traits in humans are attributed to the origin of gene duplicates with radical changes in their structure relative to the progenitor copy [26, 27, 45]. Our comparisons of the exon-intron structure of paralogs revealed that *complete* duplicates are the dominant structural category of gene duplicates stemming from DNA-mediated duplication events within the human genome. Only 17 % gene of duplicate pairs stemming from DNA-mediated duplication events comprise structurally heterogeneous duplicates.

The predominance of *complete* duplicates in the human genome is also notably different from the genomes of a handful of other multicellular eukaryotic species in which detailed structural characterization of paralogs has been conducted at a genome-wide scale [13, 15, 16]. The high frequency of *complete* duplicates in the human genome is especially intriguing given that the length of human protein-coding genes is quite substantial with a mean and median length of 65 and 25 kb, respectively. Because the duplication machinery is expected to be impervious to gene boundaries, the likelihood of capturing an entire ORF during duplication is more likely in compact genomes with a shorter average gene length [3]. Given the larger genome size and average gene length in humans relative to worm and *Drosophila*, it is paradoxical that *complete* duplicates represent the most abundant structural class of gene duplicates within the human genome. However, our investigation into the distribution of duplication spans of human paralogs may provide some insight regards this paradox. The median duplication span in our data set is significantly greater than the median gene length in humans. Hence, the high prevalence of *complete* duplication events within our data set of young human gene duplicates may be explained by human duplicons having lengthier tracts, although the role of purifying selection against shorter duplication tracts yielding *partial* and *chimeric* duplicates cannot be ruled out. However, with increasing evolutionary age, we observed a significant increase in the frequency of both *partial* and *chimeric* duplicates as well as a concomitant attenuation of duplication spans. This noticeable decline in the frequency of *complete* duplicates with increasing evolutionary age is in stark contrast to the pattern observed in macaques, orang-utans and chimpanzees wherein the ratio of *complete*/*partial* gene duplications increased as a function of evolutionary age [19]. The observed increase in the frequency of *partial* and *chimeric* duplicates with evolutionary age has two explanations, namely (i) enhanced survivorship of *partial* and *chimeric* duplicates and/or stronger selection against *complete* duplicates, and/or (ii) gene rearrangements or deletion events that serve to erode the sequences of lengthier, *complete* duplicates and thereby reduce their detectable duplication spans. This also implies that our observed median duplication span is likely a conservative estimate, given the possibility of sequence decay in older duplicates.

The large fraction of *complete* duplicates within our data set begs the question as to how the majority of newly minted human duplicate genes are able to rapidly assume unique species-specific functions. While the relationship between structural category of duplicates and signatures of accelerated evolution has not been conducted at a genome-wide scale in humans, there is some evidence to suggest that human paralogs can diverge rapidly. Zhang et al. [46] found that for a large fraction of putatively young human paralogs (*K*_*S*_ < 0.3), one copy exhibited a signature of rapid molecular evolution at the amino-acid level and less stringent selective constraints (high *K*_*A*_/*K*_*S*_ ratios). Makova and Li [47] demonstrated diverged spatial expression profiles for a large proportion of human paralogs, noting that the expression divergence increased approximately linearly with evolutionary time (*K*_*S*_). In a study of the expression of *complete* gene duplications in six tissues in humans and nonhuman primates, Gokcumen et al. [19] found that the emergence of new *complete* duplicates often coincides with gene expression in new tissues. In a similar vein, analysis of a human gene coexpression network revealed that even evolutionarily young gene duplicates rapidly gained new coexpressed partners [48]. Studies of the patterns of sequence and functional divergence between human paralogs can be further elucidated by future investigations into whether, and the extent to which, structural resemblance between paralogs impinges on the evolution of novel function. Is the evolution of novel function primarily facilitated by changes to the intron-exon structure of the derived copy relative to its progenitor as manifest in *partial* and *chimeric* duplicates or do regulatory changes (rapid promoter evolution or the gain of novel promoters) play a significant role?

89 % of genes duplicates within our data set bear signatures of origin from DNA-mediated events. This genomic proximity between paralogs suggests a major role for slippage and unequal exchange as major mutational mechanisms in the creation of human gene duplicates. Non-allelic homologous recombination (NAHR) and non-homologous end joining (NHEJ) are two mechanisms of double-strand break repair that are implicated as common mutational mechanisms for the origin of gene duplicates. While we did not conduct sequence analysis of breakpoint junctions of paralogs within our data set to distinguish their relative contributions, both mechanisms likely contributed to the formation of gene duplicates from DNA-mediated events in our data set. The relative contributions of NAHR and NHEJ in generating structural variants in humans and other nonhuman primates is still under debate, with some studies favoring NAHR as the dominant mutational mechanism in the creation of copy-number variation (including duplications) [19, 49] and others implicating NHEJ in the creation of human structural variation across the genome [50] and in the origin of segmental duplications in human subtelomeric regions [51].

The vast majority of gene duplicates in our data set (83 %) tend to reside on the same chromosome (*intrachromosomal* duplicates), which may implicate NAHR in their formation. With respect to *intrachromosomal* duplicates, paralogs in *inverse* transcriptional orientation are equally frequent as paralogs in *direct* orientation. Inter-cohort comparisons found no significant difference in the proportions of *direct vs. inverted intrachromosomal* paralogs with increasing evolutionary age. This pattern of transcriptional orientation of putatively young human paralogs is in direct contrast to *C. elegans*. In *C. elegans*, a significant majority of *intrachromosomal* duplicates within the *K*_*S*_ = 0 age-cohort tend to occur as adjacent loci in inverted orientation but evolutionarily older paralogs exhibit roughly equal proportions of *inverse vs. direct* orientation [13]. Hence, humans appear to have a lower proportion of inverted duplications at birth than *C. elegans*. The results suggest that *direct* paralogs in the human genome are equally stable as *inverted* duplicates and local-scale inversion events do not play a major role in secondary movement or switching of transcriptional orientation with the progression of evolutionary time.

Studies of gene duplicates in eukaryotic genomes have detected an increase in distance between paralogs with increasing age (*K*_*S*_), a trend frequently ascribed to secondary movement of genes [52, 53]. That is, the derived, duplicated locus originates in close proximity to the ancestral locus and at some later point in evolutionary time, secondary gene rearrangements lead one or both paralogs to new and more distant genomic locations. This ‘secondary movement’ hypothesis, if true, would be manifest as a positive relationship between *K*_*S*_ and genomic distance. However, this positive correlation between duplicate age and genomic distance could also be explained by the differential survival of paralogs. The loss of duplicate genes may be facilitated by their proximity, for instance, by more frequent unequal crossing-over between closely-spaced paralogs. However, we did not find a significant correlation between *K*_*S*_ and the distance between extant *intrachromosomal* paralogs suggesting that (i) paralogs on the same chromosome do not migrate away from each other with evolutionary time, and (ii) nor do closer-spaced *intrachromosomal* paralogs suffer a higher loss rate. In contrast, we find that the average age of *interchromosomal* paralogs is higher than that of *intrachromosomal* paralogs and the difference is still significant even when we exclude RNA-mediated duplications (characterized by high *K*_*S*_ values and occurrence on different chromosomes). All 39 duplicate pairs in the *K*_*S*_ = 0 cohort are *intrachromosomal*, suggesting that new duplicates in the human genome overwhelmingly originate on the same chromosome as the parental copy, a pattern similar to that in *C. elegans* and *Drosophila melanogaster* [13, 14] but in contrast to small segmental duplications in *S. cerevisiae* [15]. The findings that evolutionarily older gene duplicates possess higher proportions of *interchromosomal* duplicates and a lack of association between distance and *K*_*S*_ among *intrachromosomal* paralogs is similar to a previous result in *C. elegans* [13].

The chromosomal distribution of young gene duplicates can elucidate whether there exist certain mutational hotspots for their origin with respect to specific chromosomes as well as locations along the gradient of a chromosome. Regards chromosomal location, the distribution of gene duplicates on autosomes did not differ significantly from a random distribution, after normalizing for chromosome-specific gene density. Hence, the probability of a gene duplication or retention of gene duplicates does not appear to differ between the autosomes. However, there was an abundance of gene duplicates on the sex chromosomes (three- and 17-fold on the X and Y chromosomes, respectively), after accounting for the density of protein-coding genes. It is possible that the duplication rates are higher on the sex chromosomes than the autosomes, or the retention of sex-linked gene duplicates is higher (lower loss rate). The abundance of putative young gene duplicates on the Y chromosome is notable given that it is an especially gene depauperate environment. With respect to the location of gene paralogs along chromosomes, we found evidence for spatial clustering of duplicates with centromeric regions exhibiting a significant excess of gene duplicates. This nonrandom, pericentromeric gradient of duplications in the human genome has been noted by preceding studies of rodent paralogs [54], human gene duplicates on Chromosome 22 [55] as well as at a genome-wide scale [18, 49, 50, 56, 57]. This pattern, moreover, is not restricted to humanoids. Emerson et al. [58] observed an enrichment of duplications in pericentromeric regions in a population-genomic study of CNVs in 15 isofemale *D. melanogaster* lines.

Although, DNA-mediated events are responsible for the origin of the vast majority of young gene duplicates in the human genome, we identified ~11 % of duplicates as putatively originating from RNA-mediated events. These putative retroduplicates pairs possessed several key diagnostic characteristics that implicate retrotransposition. The age distribution of putative retroposed human gene duplicates presented an interesting pattern, displaying increased frequencies with increasing evolutionary age (*K*_*S*_), and a complete absence of retroposed duplicates in the *K*_*S*_ = 0 age-cohort. Although the small sample size of retroposed duplicates within our data set precludes a robust explanation for this trend, we speculate that this pattern may represent a burst of retrotranspositional activity yielding gene duplicates in our species’ recent evolutionary past.

## Conclusions

Our analyses of putative young gene duplicates in the human genome have revealed both similarities and differences with other species. As in *C. elegans*, there is a significant increase in the proportion of *interchromosomal* paralogs with increasing evolutionary age, but without a similar increase in distance with age within *intrachromosomal* paralogs. Young human paralogs differ in some other aspects from their counterparts in *C. elegans* and *Drosophila*. For instance, inverted duplications are less common among the most recent paralogs in humans than in *C. elegans* [13], but their proportions are stable with age. This may indicate differences in prevailing duplication and duplication loss mechanisms in these species. In addition, human duplicates have, on average, much larger duplication spans which are more likely to capture entire ORFs leading to *complete* duplicates compared to higher proportions of structurally heterogeneous duplicates (*partial* and *chimeric* duplications) in *Drosophila* and *C. elegans*. The change in the genomic and structural features of human paralogs with evolutionary time demonstrate that (i) genomic context and structural similarities have important consequences for the fate of duplicate genes, and (ii) the mutational spectrum of gene duplicates and their subsequent evolutionary dynamics can vary significantly among eukaryotic species.

## Methods

### Similarity based grouping and estimation of evolutionary divergence

Genome sequences and annotated genome features for the human genome assembly GRCh37 were downloaded from Ensembl release version 72 [59]. To minimize the inclusion of splice variants during the similarity search, we selected the longest transcript for each coding gene as the canonical transcript using in-house Perl scripts. Protein sequences and coding sequences of 20,214 canonical transcripts were downloaded from the BioMart interface of the Ensembl site. Similarity search was performed using an all-against-all BLASTP with a cut-off E-value of ≤ 10^−10^ and an amino acid identity ≥ 40 %. To ensure that evolutionarily young but structurally heterogeneous gene duplicates (e.g. *partial* or *chimeric* duplicates) were not excluded from the initial sequence filtration steps, we did not use the high identity cut-off of 90 %, which is widely used in other studies of this nature. Genes with higher levels of similarity than the cut-off value were clustered into one family. Multiple genes were pooled into one gene family based on the single-link principle. For example, if protein A hits proteins B and C with BLASTP E-values ≤ 10^−10^ and identity ≥ 40 %, then A, B, and C were included in the same family, regardless of the similarity for the comparison of B and C. Linked duplicate sets, which comprised the duplication of multiple open reading frames via a single duplication event, were treated as a single gene duplicate. The *K*_*S*_ values of all members within a linked set were averaged to yield a single *K*_*S*_ value.

For each gene duplicate pair, a protein sequence alignment was generated by the CLUSTALW2 program [60]. Thereafter, the nucleotide sequences were aligned based on the protein sequence alignment profile using PAL2NAL [61]. The measure of synonymous sequence divergence in coding regions (*K*_*S*_) for gene paralogs was recalculated using the pairwise model (runmode = −2) of the *codeml* program in the PAML package [62]. Putative evolutionarily young gene duplicate pairs (*K*_*S*_ ≤ 0.1) were retained for further analysis.

### Investigating the frequency of ectopic gene conversion between paralogous sequences

For each of the 184 duplicate pairs within our dataset, protein sequences of both human paralogs were used as queries in the BLASTP program to search and identify, where possible, the best hit in the chimpanzee protein database. The coding sequences of the human paralogs and their best-hit chimpanzee ortholog (s) were input and aligned in a single sequence file using the CLUSTALW2 program [60]. A statistical test for gene conversion was implemented in the GENECONV program, version 1.81a [63] with default settings and additional option (/lp) to detect both global and pairwise inner fragments supporting gene conversion. Significance of gene conversion was determined by a permutation test correcting for multiple comparisons.

### Visualization of duplication breakpoints and determination of the degree of structural resemblance between paralogs

To locate the duplication breakpoints for large human gene pairs, sequences within 200 kb flanking region (800 kb for few pairs) of each gene were aligned using the pairwise alignment tool LASTZ [64]. The LASTZ program uses a seeded pattern-matching method to find out local similarities for large genomic DNA sequences. To obtain a graphic view for all identified young gene duplicates, the LASTZ alignment results in conjunction with the genome features were imported into the Generic Synteny Browser, GBrowse_syn [65]. With the aid of an interactive alignment of the two focal paralogous sequences, we further identified the duplication break points, duplication span, and the degree of structural resemblance between paralogs [13].

We further filtered out *same-location* pairs and *shadow/redundancy* pairs for gene families comprising three to five members. The *same-location* pairs shared the same chromosomal coordinates while being assigned different gene names. This was taken to reflect annotation errors rather than true gene duplication events. We also removed *shadow* pairs within multiple-member gene families, which were representative of sequence similarity rather than true duplication events. For example, a five-member gene family could have been generated through four gene duplication events, although BLASTP would yield ten gene duplicates pairs based on pairwise comparisons of sequence similarity. In this hypothetical example, only four gene duplicate pairs representing the true duplication events were retained, while removing the six additional duplicate pairs displaying sequence similarity. The representative four gene duplicate pairs were selected for inclusion based on a UPGMA tree generated from their pairwise *K*_*S*_ values.

The initial genome-wide search identified 286 gene duplicates pairs with low synonymous divergence in the human genome based on DNA (or protein) sequence similarity. The putative gene duplicates were subsequently filtered with respect to evolutionary age (*K*_*S*_ ≤ 0.1) and family size (≤5 members). During the visualization check, 24 *same-location* pairs and 57 *shadow* pairs were removed, and 64 gene pairs were merged into 42 linked sets. Finally, we identified 184 duplication events, comprising 142 non-linked duplications and 42 linked sets.

### Statistical tests

Statistical tests were performed using the R program package version 3.01 [66]. All duplicate pairs were initially classified into three age-cohorts (*K*_*S*_ = 0, 0 < *K*_*S*_ ≤ 0.025, and 0.025 < *K*_*S*_ ≤ 0.1). If the latter two of the three cohorts showed no significant statistical difference with respect to the focal characteristic, comparisons were then performed between two cohorts (*K*_*S*_ = 0, and 0 < *K*_*S*_ ≤ 0.1).

### Chromosomal location

The frequency distribution of duplications between and within chromosomes was analyzed with a goodness-of-fit *G*-test. The number of gene duplicates per chromosome was compared to the number of protein-coding genes per chromosome. Each gene duplicate pair with both paralogs residing on the same chromosome was counted as a single duplication event. In instances where the two paralogs were located on different chromosomes, each paralog was counted as a half event. This was done because both paralogs resulted from a single duplication event and the identity and location of the ancestral paralog could not be determined. A goodness-of-fit test was also performed on the distance of *intrachromosomal *paralogs from the centromeres. The chromosomes were divided into 10 Mb bins and the number of duplicates compared to the number of genes per bin. In the events that the two paralogs comprising a duplicate pair were located in different bins, each paralog was counted as half.

### Availability of data and materials

The data sets supporting the results of this article are included with the article as supplementary material.

## Additional files

Additional file 1: Table S1. Evolutionary and genomic features of 184 gene duplicates with low synonymous divergence in the human genome. (PDF 262 kb) Additional file 2: Figure S1. Decrease in the frequency of *intrachromosomal* duplicates with *K*_*S*_ in three age-cohorts. **Figure S2.** The physical distance between *intrachromosomal* paralogs as a function of *K*_*S*_. **Figure S3.** Duplication span of DNA- and RNA-mediated duplicates as a function of *K*_*S*_. **Figure S4.** Transcriptional orientation (*direct* versus *inverse*) of *intrachromosomal* duplicates within three age-cohorts. **Figure S5.** Frequencies of three structural categories of DNA-mediated gene duplicates across three age-cohorts. **Figure S6.** Box plot displaying the distribution of minimum duplication span for young gene duplicates. (PDF 303 kb)
