# Supplementary material for: Early evolutionary history and genomic features of gene duplicates in the human genome
Source: BMC Genomics. 2015 Aug 20;16(1):621. doi: 10.1186/s12864-015-1827-3 (PMC4546093; doi:10.1186/s12864-015-1827-3)
Supplement: Additional file 1: Table S1. — Evolutionary and genomic features of 184 gene duplicates with low synonymous divergence in the human genome. (PDF 262 kb) [file 12864_2015_1827_MOESM1_ESM.pdf]

Supplementary Table S1. Evolutionary and genomic features of 184 gene duplicates with low synonymous divergence in the human genome. Accession numbers in columns 1 and 2 correspond to Ensembl ID version 72 released in June 2013. Column 3 presents the synonymous sequence divergence (K_S) between the two paralogs as computed by maximum likelihood. Column 4 presents the nonsynonymous sequence divergence (K_A) between the two paralogs as computed by maximum likelihood. Column 5 presents the K_A/ K_S ratio. Chromosomal location of the two paralogs, physical distance and strand orientation if present on the same chromosome are presented in columns 6, 7, and 8, respectively. Column 9 lists the particular category of structural resemblance between the two paralogs. Structural resemblance between paralogs within a duplicate pair was classified as (i) *complete* if sequence homology between the focal paralogs extended throughout their open reading frames (ORFs) from the start to the stop codon and possibly extending into one or both flanking regions, (ii) *partial* if one paralog possessed unique exon(s) in its ORF that are absent in the other paralog, (iii) *chimeric* if both paralogs contained unique exon(s) and/or intron(s) within their respective ORFs to the exclusion of the other paralog, and (iv) *retroposed* if the ORF of one paralog contained one or more intron(s) which were absent in the other paralog's ORF. Column 10 provides an estimate of the length of the duplicated region. Column 11 identifies particualr gene duplicate pairs that were duplicated with other loci as linked sets.

Paralog A Ensemble Gene ID	***Paralog B Ensemble Gene ID***	K_S	K_A	K_A/K_S	***Chromosomal Location***	***Physical Distance (bp)***	***Transcriptional Orientation***	***Structural Category***	***Duplication Span (bp)***	***Linked?***
ENSG00000232948	ENSG00000233050	0.0000	0.0000	0.505	8/8	147895	-/-	Complete	98670	
ENSG00000230000	ENSG00000268181	0.0000	0.0000	0.413	7/7	8473	+/-	Complete	147994	
ENSG00000182646	ENSG00000179304	0.0000	0.0000	0.522	X/X	9934	-/+	Complete	44513	
ENSG00000215033	ENSG00000215020	0.0000	0.0033	99.000	10/10	418855	+/+	Complete	88553	
ENSG00000269358	ENSG00000269831	0.0001	0.0121	99.000	1/1	223324882	-/-	Complete	152319	
ENSG00000197077	ENSG00000251180	0.0007	0.0000	0.001	22/GL000242.1	NA	+/+	Partial	43479	
ENSG00000183474	ENSG00000145736	0.0036	0.0034	0.935	5/5	1362518	+/-	Complete	95695	
ENSG00000205595	ENSG00000109321	0.0042	0.0000	0.001	4/4	128793	+/+	Complete	40981	
ENSG00000168028	ENSG00000205246	0.0049	0.0045	0.906	3/19	NA	+/+	Retroposed	2527	
ENSG00000204661	ENSG00000228259	0.0060	0.0656	10.983	5/5	7724	-/+	Partial	6069	
ENSG00000197620	ENSG00000197021	0.0076	0.0101	1.334	X/X	441777	+/-	Complete	29776	
ENSG00000182356	ENSG00000239511	0.0096	0.0084	0.876	22/22	2614973	+/-	Complete	38208	
ENSG00000205076	ENSG00000178934	0.0152	0.0000	0.001	19/19	8415	-/+	Complete	7597	
ENSG00000184945	ENSG00000185176	0.0157	0.0044	0.281	2/2	3178	+/-	Complete	13058	
ENSG00000105835	ENSG00000229644	0.0215	0.0029	0.137	7/10	NA	-/-	Retroposed	2701	
ENSG00000204936	ENSG00000204933	0.0231	0.0110	0.475	19/19	3228	+/-	Partial	29644	
ENSG00000186825	ENSG00000197927	0.0291	0.0290	0.997	2/2	19789	-/+	Chimeric	9690	

ENSG00000122852	ENSG00000185303	0.0294	0.0167	0.568	10/10	34380	+/-	Complete	11159	
ENSG00000269337	ENSG00000268578	0.0300	0.0000	0.001	9/9	25025042	+/-	Complete	151099	
ENSG00000143185	ENSG00000143184	0.0308	0.0084	0.274	1/1	27416	-/+	Complete	6164	
ENSG00000148672	ENSG00000182890	0.0318	0.0220	0.692	10/X	NA	-/+	Retroposed	3180	
ENSG00000115042	ENSG00000144199	0.0323	0.0098	0.303	2/2	1668109	+/-	Complete	14879	
ENSG00000269099	ENSG00000130592	0.0326	0.0102	0.313	13/11	NA	+/+	Partial	136	
ENSG00000175548	ENSG00000139133	0.0330	0.0250	0.755	12/12	4364951	+/+	Complete	152707	
ENSG00000196459	ENSG00000256060	0.0451	0.0000	0.001	X/19	NA	-/+	Retroposed	2680	
ENSG00000229924	ENSG00000171847	0.0464	0.0335	0.722	4/12	NA	+/-	Complete	233500	
ENSG00000114547	ENSG00000065371	0.0472	0.0228	0.484	3/3	1917158	+/-	Complete	61111	
ENSG00000033011	ENSG00000189366	0.0529	0.0384	0.725	16/3	NA	+/-	Partial	24702	
ENSG00000128185	ENSG00000183628	0.0542	0.0224	0.414	22/22	1376928	-/+	Complete	21921	
ENSG00000169469	ENSG00000169474	0.0554	0.0330	0.595	1/1	44865	+/+	Complete	2205	
ENSG00000099721	ENSG00000125363	0.0564	0.0725	1.286	Y/X	NA	-/+	Complete	10875	
ENSG00000188672	ENSG00000187010	0.0660	0.1021	1.546	1/1	33395	-/+	Complete	61003	
ENSG00000110057	ENSG00000233094	0.0707	0.0205	0.289	11/GL000222.1	NA	-/+	Partial	53411	
ENSG00000212643	ENSG00000169249	0.0724	0.0294	0.406	5/X	NA	+/+	Retroposed	3083	
ENSG00000253797	ENSG00000156697	0.0780	0.0489	0.627	13/X	NA	+/+	Retroposed	2481	
ENSG00000178700	ENSG00000228716	0.0785	0.0452	0.576	3/5	NA	-/-	Retroposed	3499	
ENSG00000229314	ENSG00000228278	0.0793	0.0476	0.600	9/9	1	+/+	Complete	7053	
ENSG00000166926	ENSG00000110077	0.0817	0.1230	1.505	11/11	152178	+/-	Partial	7898	
ENSG00000243709	ENSG00000143768	0.0851	0.0210	0.247	1/1	48103	-/-	Complete	2611	
ENSG00000243317	ENSG00000267889	0.0858	0.0199	0.232	7/2	NA	+/+	Retroposed	848	
ENSG00000196659	ENSG00000197557	0.0876	0.0247	0.282	2/2	59754	-/-	Complete	5829	
ENSG00000213714	ENSG00000124103	0.0896	0.0698	0.778	20/20	3416	+/+	Complete	4416	
ENSG00000130741	ENSG00000180574	0.0932	0.0445	0.478	X/12	NA	+/+	Retroposed	2925	
ENSG00000172115	ENSG00000269383	0.0982	0.1541	1.569	7/2	NA	-/-	Complete	1210	
ENSG00000224389	ENSG00000244731	0.0025	0.0025	1.019	6/6	1	+/+	Complete	32741	
ENSG00000122543	ENSG00000135175	0.0259	0.0101	0.390	7/7	91655248	+/-	Complete	51956	
ENSG00000185897	ENSG00000126251	0.0001	0.0076	99.000	19/19	9518	+/+	Complete	2936	
ENSG00000253626	ENSG00000132507	0.0175	0.0088	0.505	10/17	NA	+/+	Retroposed	1134	
ENSG00000187630	ENSG00000157326	0.0000	0.0000	0.434	14/14	1	+/+	Complete	35350	
ENSG00000269011	ENSG00000268851	0.0000	0.0000	0.001	21/GL000215.1	NA	-/-	Complete	172472	
ENSG00000170074	ENSG00000204677	0.0001	0.0089	99.000	5/5	217864	-/+	Complete	51458	
ENSG00000072444	ENSG00000204147	0.0001	0.0057	99.000	10/10	4485559	-/+	Partial	43204	
ENSG00000188611	ENSG00000204147	0.0000	0.0031	99.000	10/10	540267	-/+	Partial	43204	
ENSG00000143556	ENSG00000184330	0.0747	0.0316	0.423	1/1	16018	-/+	Complete	24384	

ENSG00000122696	ENSG00000141437	0.0323	0.0214	0.662	9/18	NA	-/-	Retroposed	1272	
ENSG00000212899	ENSG00000212900	0.0688	0.0088	0.127	17/17	5142	-/-	Complete	605	
ENSG00000253506	ENSG00000196531	0.0728	0.0676	0.929	17/12	NA	-/-	Retroposed	836	
ENSG00000197110	ENSG00000183709	0.0207	0.0191	0.922	19/19	13699	-/+	Complete	7838	
ENSG00000149531	ENSG00000220023	0.0226	0.0477	2.112	20/GL000219.1	NA	+/-	Partial	73153	
ENSG00000188092	ENSG00000117262	0.0037	0.0000	0.001	1/1	1561389	+/-	Complete	85051	
ENSG00000157322	ENSG00000157335	0.0104	0.0030	0.285	16/16	157874	+/+	Complete	45147	
ENSG00000157335	ENSG00000140839	0.0034	0.0050	1.443	16/16	4188011	+/-	Complete	39139	
ENSG00000184814	ENSG00000233701	0.0951	0.0770	0.810	3/3	21031	-/-	Complete	2791	
ENSG00000184814	ENSG00000206260	0.0657	0.0492	0.748	3/3	12807	-/-	Complete	1622	
ENSG00000204397	ENSG00000255221	0.0918	0.0886	0.965	11/11	49331	-/-	Partial	6535	
ENSG00000171102	ENSG00000122136	0.0090	0.0439	4.851	9/9	2290487	-/+	Complete	34319	
ENSG00000184033	ENSG00000183678	0.0000	0.0000	53.484	X/X	21622	-/+	Complete	35968	
ENSG00000197665	ENSG00000228157	0.0078	0.0040	0.511	17/17	45996	-/+	Complete	45009	
ENSG00000228157	ENSG00000230493	0.0078	0.0040	0.511	17/17	72764	+/+	Complete	53320	
ENSG00000182824	ENSG00000188280	0.0515	0.0264	0.513	22/22	1945636	+/+	Chimeric	26142	
ENSG00000240247	ENSG00000239839	0.0000	0.0049	99.000	8/8	1	-/-	Complete	19104	
ENSG00000240247	ENSG00000206047	0.0000	0.0000	0.001	8/8	1	-/-	Complete	19104	
ENSG00000177710	ENSG00000164729	0.0237	0.0401	1.687	8/17	NA	+/-	Complete	3057	
ENSG00000164729	ENSG00000259224	0.0242	0.0412	1.701	17/17	26133000	-/+	Retroposed	1696	
ENSG00000254598	ENSG00000101266	0.0970	0.0548	0.565	11/20	NA	-/-	Retroposed	1534	
ENSG00000166157	ENSG00000132958	0.0752	0.0881	1.173	21/13	NA	-/-	Complete	94110	
ENSG00000170215	ENSG00000154537	0.0209	0.0000	0.001	9/9	22291902	-/+	Complete	284036	
ENSG00000268942	ENSG00000173207	0.0503	0.0497	0.989	5/1	NA	-/+	Retroposed	1746	
ENSG00000173432	ENSG00000134339	0.0591	0.0766	1.296	11/11	15647	+/-	Partial	4464	
ENSG00000196312	ENSG00000148110	0.0834	0.0732	0.879	9/9	2510563	-/+	Chimeric	78167	
ENSG00000099984	ENSG00000133433	0.0115	0.0018	0.161	22/22	2128	+/-	Complete	30285	
ENSG00000186523	ENSG00000118894	0.0880	0.0388	0.441	8/16	NA	-/-	Complete	49398	
ENSG00000237847	ENSG00000268991	0.0000	0.0000	0.432	1/1	44330	+/-	Complete	35034	
ENSG00000203815	ENSG00000268991	0.0526	0.0549	1.043	1/1	132612981	+/-	Complete	1684	
ENSG00000203815	ENSG00000268674	0.0635	0.0911	1.435	1/1	132808823	+/+	Complete	1686	
ENSG00000099290	ENSG00000172661	0.0114	0.0062	0.548	10/10	5503229	+/+	Complete	101659	
ENSG00000226784	ENSG00000171314	0.0093	0.0211	2.266	X/10	NA	-/+	Retroposed	1689	
ENSG00000212724	ENSG00000213417	0.0376	0.0000	0.001	17/17	5218	-/-	Complete	576	
ENSG00000214518	ENSG00000212725	0.0000	0.0000	0.303	17/17	7362	-/-	Complete	582	
ENSG00000212725	ENSG00000213417	0.0000	0.0029	99.000	17/17	17932	-/-	Complete	661	
ENSG00000174428	ENSG00000196275	0.0011	0.0037	3.427	7/7	203874	+/-	Complete	143572	

ENSG00000163283	ENSG00000163286	0.0632	0.0140	0.221	2/2	18456	+/+	Complete	8712	
ENSG00000238083	ENSG00000176681	0.0040	0.0053	1.337	17/17	19900	+/+	Complete	197813	
ENSG00000176681	ENSG00000176809	0.0478	0.0285	0.595	17/17	18413859	+/-	Complete	66453	
ENSG00000139223	ENSG00000140350	0.0783	0.0629	0.804	12/15	NA	+/-	Retroposed	646	
ENSG00000169763	ENSG00000169807	0.0000	0.0000	0.461	Y/Y	1441567	-/-	Complete	168120	
ENSG00000269526	ENSG00000268964	0.0345	0.0239	0.692	19/19	1	+/+	Complete	32741	
ENSG00000143954	ENSG00000172016	0.0973	0.0736	0.757	2/2	127878	+/-	Complete	3641	
ENSG00000205456	ENSG00000205457	0.0071	0.0000	0.001	16/16	634356	+/+	Complete	306492	
ENSG00000205456	ENSG00000261509	0.0071	0.0000	0.001	16/16	865729	+/+	Complete	130935	
ENSG00000205457	ENSG00000183632	0.0000	0.0000	0.384	16/16	214228	+/-	Complete	183089	
ENSG00000203817	ENSG00000215784	0.0001	0.0057	99.000	1/1	5411200	-/-	Partial	35229	
ENSG00000188610	ENSG00000215784	0.0065	0.0142	2.168	1/1	23007885	+/-	Complete	124962	
ENSG00000111775	ENSG00000226976	0.0219	0.0087	0.396	12/6	NA	+/+	Retroposed	553	
ENSG00000227151	ENSG00000229665	0.0000	0.0000	0.399	13/13	1	+/+	Complete	27725	
ENSG00000227151	ENSG00000234278	0.0000	0.0000	0.001	13/13	1	+/+	Complete	27725	
ENSG00000204918	ENSG00000229665	0.0000	0.0000	0.001	13/13	1	+/+	Complete	27725	
ENSG00000204918	ENSG00000204919	0.0000	0.0000	0.440	13/13	1	+/+	Complete	27725	
ENSG00000174876	ENSG00000187733	0.0000	0.0000	0.470	1/1	19160	-/+	Complete	34804	
ENSG00000174876	ENSG00000240038	0.0724	0.0128	0.177	1/1	107727	-/+	Complete	8790	
ENSG00000237763	ENSG00000187733	0.0000	0.0000	0.059	1/1	45596	+/+	Complete	48555	
ENSG00000243480	ENSG00000240038	0.0550	0.0063	0.114	1/1	37007	+/+	Complete	8829	
ENSG00000196507	ENSG00000204071	0.0708	0.0383	0.541	X/X	1464257	+/-	Complete	3441	
ENSG00000183461	ENSG00000204375	0.0000	0.0000	0.001	X/X	21643	+/-	Complete	28680	
ENSG00000244067	ENSG00000182793	0.0993	0.0667	0.672	6/6	63506	-/-	Complete	17783	
ENSG00000244067	ENSG00000243955	0.0582	0.0257	0.442	6/6	19782	-/-	Complete	20881	
ENSG00000228532	ENSG00000188612	0.0198	0.0057	0.290	X/17	NA	-/-	Retroposed	2379	
ENSG00000177688	ENSG00000188612	0.0699	0.0785	1.122	6/17	NA	+/-	Retroposed	1083	
ENSG00000220903	ENSG00000225899	0.0379	0.0267	0.703	GL000222.1/10	NA	-/-	Complete	6409	
ENSG00000220903	ENSG00000172969	0.0000	0.0000	0.477	GL000222.1/3	NA	-/+	Complete	186774	
ENSG00000205097	ENSG00000148828	0.0000	0.0000	0.425	4/GL000228.1	NA	-/-	Complete	98341	
ENSG00000225899	ENSG00000148828	0.0037	0.0072	1.922	10/GL000228.1	NA	-/-	Complete	74632	
ENSG00000169953	ENSG00000172468	0.0000	0.0000	0.001	Y/Y	39643	-/+	Complete	190162	
ENSG00000185554	ENSG00000185945	0.0016	0.0000	0.001	X/X	10765	+/-	Complete	140562	
ENSG00000237289	ENSG00000223572	0.0046	0.0000	0.001	15/15	1	+/+	Complete	92931	
ENSG00000105889	ENSG00000164647	0.0062	0.0258	4.160	7/7	67145303	-/+	Chimeric	48968	
ENSG00000196934	ENSG00000183246	0.0000	0.0005	99.000	22/22	48673	+/-	Complete	70268	
ENSG00000196622	ENSG00000183246	0.0008	0.0033	3.924	22/22	1344155	-/-	Complete	99520	

ENSG00000205810	ENSG00000205809	0.0813	0.1096	1.348	12/12	4501	-/-	Partial	10755	
ENSG00000206181	ENSG00000266996	0.0889	0.0865	0.973	18/18	12842	-/-	Complete	3813	
ENSG00000266996	ENSG00000234298	0.0000	0.0017	99.000	18/18	931	-/-	Complete	4992	
ENSG00000234298	ENSG00000183791	0.0000	0.0018	99.000	18/18	923	-/-	Complete	4963	
ENSG00000223524	ENSG00000255940	0.0000	0.0000	0.001	8/8	2970	+/-	Complete	14283	
ENSG00000223524	ENSG00000205176	0.0072	0.0006	0.088	8/8	170367	+/-	Complete	3993	
ENSG00000119673	ENSG00000184227	0.0814	0.0374	0.459	14/14	1452	+/+	Complete	26573	
ENSG00000158427	ENSG00000158164	0.0377	0.0000	0.001	X/X	1426646	+/-	Complete	10733	
ENSG00000147059	ENSG00000186787	0.0038	0.0020	0.533	X/X	10452	-/-	Complete	5259	
ENSG00000136488	ENSG00000213218	0.0571	0.0035	0.061	17/17	7468	-/-	Complete	15106	
ENSG00000136488	ENSG00000259384	0.0953	0.0604	0.633	17/17	16832	-/-	Complete	4504	
ENSG00000204807	ENSG00000204804	0.0646	0.1193	1.848	9/9	1554	+/-	Complete	2935	
ENSG00000215356	ENSG00000215372	0.0206	0.0382	1.858	8/8	63872	+/-	Complete	458748	Linked Set 1
ENSG00000171711	ENSG00000177257	0.0159	0.0000	0.001	8/8	63872	+/-	Complete	Linked Set 1	
ENSG00000176797	ENSG00000177243	0.0000	0.0000	0.388	8/8	63872	+/-	Complete	Linked Set 1	
ENSG00000178287	ENSG00000164871	0.0000	0.0000	0.467	8/8	63872	+/-	Complete	Linked Set 1	
ENSG00000176782	ENSG00000177023	0.0000	0.0000	0.325	8/8	63872	+/-	Complete	Linked Set 1	
ENSG00000186579	ENSG00000187082	0.0000	0.0000	0.516	8/8	63872	+/-	Complete	Linked Set 1	
ENSG00000186562	ENSG00000186599	0.0000	0.0000	0.001	8/8	63872	-/+	Complete	Linked Set 1	
ENSG00000186572	ENSG00000198129	0.0000	0.0000	0.001	8/8	63872	-/+	Complete	Linked Set 1	
ENSG00000255378	ENSG00000255251	0.0000	0.0000	0.491	8/8	63872	-/+	Complete	Linked Set 1	
ENSG00000103226	ENSG00000103512	0.0093	0.0024	0.256	16/16	1257670	+/+	Complete	141089	Linked Set 2
ENSG00000183889	ENSG00000183426	0.0095	0.0042	0.462	16/16	1257670	+/+	Complete	Linked Set 2	
ENSG00000146574	ENSG00000122674	0.0038	0.0000	0.001	7/7	739684	-/+	Complete	99359	Linked Set 3
ENSG00000169402	ENSG00000155026	0.0017	0.0015	0.894	7/7	739684	+/-	Complete	Linked Set 3	
ENSG00000187243	ENSG00000154545	0.0000	0.0000	0.425	X/X	99855	-/+	Complete	36405	Linked Set 4
ENSG00000182776	ENSG00000179028	0.0000	0.0000	0.001	X/X	99855	-/+	Complete	Linked Set 4	
ENSG00000182230	ENSG00000170074	0.0028	0.0105	3.693	5/5	1318801	+/-	Complete	315967	Linked Set 5
ENSG00000248469	ENSG00000249109	0.0000	0.0000	0.458	5/5	1318801	-/+	Complete	Linked Set 5	
ENSG00000214967	ENSG00000214940	0.0000	0.0000	99.000	16/16	1569911	+/-	Complete	372537	Linked Set 6
ENSG00000183889	ENSG00000233024	0.0000	0.0000	0.474	16/16	1569911	+/-	Complete	Linked Set 6	
ENSG00000103226	ENSG00000185164	0.0067	0.0011	0.166	16/16	1569911	+/-	Complete	Linked Set 6	
ENSG00000174196	ENSG00000172661	0.0000	0.0000	0.001	10/10	4760447	+/+	Partial	143021	Linked Set 7
ENSG00000174194	ENSG00000188234	0.0055	0.0039	0.708	10/10	4760447	-/-	Complete	Linked Set 7	
ENSG00000233232	ENSG00000255524	0.0106	0.0318	0.816	16/16	169291	-/+	Partial	134554	Linked Set 8
ENSG00000205609	ENSG00000184110	0.0000	0.0000	0.486	16/16	169291	-/+	Complete	Linked Set 8	
ENSG00000198156	ENSG00000196993	0.0136	0.1021	0.878	16/16	169291	-/+	Complete	Linked Set 8	

ENSG00000197859	ENSG00000215616	0.0000	0.0000	0.466	9/GL000201.1	NA	+/+	Partial	36106	Linked Set 9
ENSG00000196990	ENSG00000215611	0.0181	0.0046	0.255	9/GL000201.1	NA	-/-	Complete	Linked Set 9	
ENSG00000172058	ENSG00000205572	0.0000	0.0000	0.479	5/5	381073	+/+	Complete	494845	Linked Set 10
ENSG00000172062	ENSG00000205571	0.0000	0.0145	6.722	5/5	381073	+/+	Complete	Linked Set 10	
ENSG00000184040	ENSG00000148483	0.0000	0.0000	0.393	10/10	50001	+/+	Complete	196904	Linked Set 11
ENSG00000120586	ENSG00000183748	0.0050	0.0009	0.182	10/10	50001	+/+	Complete	Linked Set 11	
ENSG00000204807	ENSG00000232833	0.0003	0.0453	3.805	9/9	21954191	+/-	Complete	145047	Linked Set 12
ENSG00000182368	ENSG00000170215	0.0000	0.0000	0.393	9/9	21954191	+/-	Complete	Linked Set 12	
ENSG00000198444	ENSG00000185990	0.0000	0.0000	0.447	X/X	66875	+/-	Complete	50563	Linked Set 13
ENSG00000198307	ENSG00000185978	0.0000	0.0000	1.355	X/X	66875	+/-	Complete	Linked Set 13	
ENSG00000267985	ENSG00000268891	0.0000	0.0000	0.468	7/7	2408306	+/-	Complete	199323	Linked Set 14
ENSG00000155428	ENSG00000178809	0.0259	0.0016	0.063	7/7	2408306	-/+	Complete	Linked Set 14	
ENSG00000196313	ENSG00000135213	0.0192	0.0291	0.730	7/7	2408306	+/-	Complete	Linked Set 14	
ENSG00000169627	ENSG00000183336	0.0000	0.0000	0.001	16/16	592983	-/-	Complete	146356	Linked Set 15
ENSG00000132207	ENSG00000181625	0.0000	0.0000	0.001	16/16	592983	+/+	Complete	Linked Set 15	
ENSG00000261052	ENSG00000213648	0.0000	0.0000	0.499	16/16	592983	+/+	Complete	Linked Set 15	
ENSG00000198064	ENSG00000169203	0.0015	0.0000	0.001	16/16	592983	-/-	Complete	Linked Set 15	
ENSG00000258150	ENSG00000258130	0.0000	0.0011	99.000	16/16	592983	-/-	Complete	Linked Set 15	
ENSG00000189266	ENSG00000215700	0.0000	0.0000	0.391	1/GL000191.1	NA	+/+	Complete	106432	Linked Set 16
ENSG00000188529	ENSG00000215699	0.0000	0.0030	99.000	1/GL000191.1	NA	-/-	Complete	Linked Set 16	
ENSG00000204149	ENSG00000174194	0.0146	0.0066	0.456	10/10	197048	+/-	Complete	181193	Linked Set 17
ENSG00000138297	ENSG00000204152	0.0001	0.0281	3.134	10/10	197048	-/+	Partial	Linked Set 17	
ENSG00000235173	ENSG00000230567	0.0000	0.0011	99.000	8/8	100001	+/+	Complete	69111	Linked Set 18
ENSG00000204775	ENSG00000170727	0.0000	0.0000	0.403	8/8	100001	-/-	Partial	Linked Set 18	
ENSG00000268531	ENSG00000268343	0.0000	0.0046	99.000	15/15	685431	+/+	Complete	321118	Linked Set 19
ENSG00000233917	ENSG00000230031	0.0000	0.0000	0.440	15/15	685431	-/-	Complete	Linked Set 19	
ENSG00000152086	ENSG00000075886	0.0317	0.0060	0.189	2/2	1080189	-/+	Complete	221851	Linked Set 20
ENSG00000152076	ENSG00000163040	0.0175	0.0085	0.484	2/2	1080189	-/+	Complete	Linked Set 20	
ENSG00000168255	ENSG00000228049	0.0747	0.4109	0.364	7/7	1	-/-	Complete	121324	Linked Set 21
ENSG00000005075	ENSG00000168255	0.0366	0.3899	0.410	7/7	1	-/-	Chimeric	Linked Set 21	
ENSG00000205233	ENSG00000189093	0.0000	0.0000	0.001	7/7	1	-/-	Complete	Linked Set 21	
ENSG00000170667	ENSG00000105808	0.0054	0.0011	0.205	7/7	1	-/-	Complete	Linked Set 21	
ENSG00000205238	ENSG00000173678	0.0000	0.0011	99.000	7/7	1	+/+	Complete	Linked Set 21	
ENSG00000237375	ENSG00000166351	0.0016	0.0027	1.628	GL000213.1/21	NA	-/+	Complete	164238	Linked Set 22
ENSG00000269725	ENSG00000269011	0.0000	0.0000	0.001	GL000213.1/21	NA	+/-	Complete	Linked Set 22	
ENSG00000152726	ENSG00000099290	0.0031	0.0015	0.472	10/10	3855364	+/+	Partial	89925	Linked Set 23
ENSG00000072444	ENSG00000188611	0.0044	0.0096	0.611	10/10	3855364	-/-	Partial	Linked Set 23	

ENSG00000222038	ENSG00000196834	0.0040	0.0029	0.720	2/2	1	+/-	Complete	159213	Linked Set 24
ENSG00000183292	ENSG00000184761	0.0266	0.2235	0.526	2/2	1	+/-	Complete	Linked Set 24	
ENSG00000188120	ENSG00000205916	0.0000	0.0025	99.000	Y/Y	1621997	-/+	Complete	421202	Linked Set 25
ENSG00000183753	ENSG00000185894	0.0000	0.0000	0.393	Y/Y	1621997	+/-	Complete	Linked Set 25	
ENSG00000183753	ENSG00000183795	0.0000	0.0000	99.000	Y/Y	1238175	+/+	Complete	395549	Linked Set 26
ENSG00000188120	ENSG00000187191	0.0117	0.0091	0.727	Y/Y	1238175	-/-	Complete	Linked Set 26	
ENSG00000185894	ENSG00000183795	0.0000	0.0000	0.422	Y/Y	2139	-/+	Complete	408818	Linked Set 27
ENSG00000205916	ENSG00000187191	0.0078	0.0056	0.711	Y/Y	2139	+/-	Complete	Linked Set 27	
ENSG00000196644	ENSG00000188092	0.0000	0.0026	99.000	1/1	1260168	+/+	Partial	281531	Linked Set 28
ENSG00000152042	ENSG00000203836	0.0081	0.0102	0.421	1/1	1260168	-/-	Complete	Linked Set 28	
ENSG00000172014	ENSG00000132498	0.0033	0.0054	1.660	9/9	26193469	+/-	Complete	440115	Linked Set 29
ENSG00000204788	ENSG00000232866	0.0073	0.0037	0.503	9/9	26193469	-/+	Complete	Linked Set 29	
ENSG00000204788	ENSG00000233434	0.0000	0.0029	99.000	9/9	1340388	-/-	Complete	114813	Linked Set 30
ENSG00000172014	ENSG00000196774	0.0017	0.0048	2.754	9/9	1340388	+/+	Complete	Linked Set 30	
ENSG00000232866	ENSG00000233434	0.0091	0.0066	0.729	9/9	24738227	+/-	Complete	88713	Linked Set 31
ENSG00000132498	ENSG00000196774	0.0015	0.0028	1.848	9/9	24738227	-/+	Complete	Linked Set 31	
ENSG00000198307	ENSG00000198082	0.0000	0.0037	99.000	X/X	487550	+/+	Complete	9515	Linked Set 32
ENSG00000198444	ENSG00000197932	0.0000	0.0000	0.403	X/X	487550	+/+	Complete	Linked Set 32	
ENSG00000185978	ENSG00000198082	0.0000	0.0037	99.000	X/X	565704	-/+	Complete	9515	Linked Set 33
ENSG00000185990	ENSG00000197932	0.0000	0.0000	0.405	X/X	565704	-/+	Complete	Linked Set 33	
ENSG00000172283	ENSG00000169763	0.0000	0.0000	0.001	Y/Y	842578	+/-	Complete	1054777	Linked Set 34
ENSG00000269393	ENSG00000267935	0.0000	0.0000	0.001	Y/Y	842578	-/+	Complete	Linked Set 34	
ENSG00000172288	ENSG00000172352	0.0000	0.0000	0.001	Y/Y	842578	+/-	Complete	Linked Set 34	
ENSG00000169789	ENSG00000169807	0.0000	0.0000	0.379	Y/Y	168948	+/-	Complete	283838	Linked Set 35
ENSG00000226941	ENSG00000169800	0.0031	0.0000	0.001	Y/Y	168948	+/-	Complete	Linked Set 35	
ENSG00000171928	ENSG00000175106	0.0320	0.0400	0.692	17/17	2872263	+/-	Partial	195489	Linked Set 36
ENSG00000171931	ENSG00000251537	0.0674	0.1966	0.552	17/17	2872263	+/-	Complete	Linked Set 36	
ENSG00000108448	ENSG00000251537	0.0528	0.2444	0.431	17/17	2872263	+/-	Complete	Linked Set 36	
ENSG00000249459	ENSG00000187607	0.0184	0.1020	0.636	17/17	2872263	-/+	Complete	Linked Set 36	
ENSG00000189375	ENSG00000214946	0.0553	0.0778	0.361	17/17	2872263	-/+	Complete	Linked Set 36	
ENSG00000187559	ENSG00000204793	0.0000	0.0022	99.000	9/9	1562677	+/-	Complete	189814	Linked Set 37
ENSG00000196873	ENSG00000204790	0.0068	0.0034	0.504	9/9	1562677	+/-	Complete	Linked Set 37	
ENSG00000228537	ENSG00000196400	0.0148	0.0000	0.001	9/9	1562677	-/+	Complete	Linked Set 37	
ENSG00000196873	ENSG00000136682	0.0164	0.0105	0.641	9/2	NA	+/+	Complete	149372	Linked Set 38
ENSG00000228537	ENSG00000238091	0.0000	0.0000	0.303	9/2	NA	-/-	Complete	Linked Set 38	
ENSG00000187559	ENSG00000184492	0.0216	0.1924	0.529	9/2	NA	+/+	Complete	Linked Set 38	
ENSG00000171129	ENSG00000171116	0.0000	0.0000	0.398	X/X	164854	-/+	Complete	29139	Linked Set 39

ENSG00000123584	ENSG00000166008	0.0000	0.0000	0.411	X/X	164854	-/+	Complete	Linked Set 39	
ENSG00000231997	ENSG00000204804	0.0984	0.1482	0.780	9/9	486881	+/-	Complete	152361	Linked Set 40
ENSG00000237198	ENSG00000204807	0.0001	0.0115	99.000	9/9	486881	-/+	Complete	Linked Set 40	
ENSG00000234295	ENSG00000157423	0.0051	0.0021	0.416	GL000192.1/16	NA	-/-	Partial	357293	Linked Set 41
ENSG00000215642	ENSG00000157423	0.0050	0.0049	0.970	GL000192.1/16	NA	-/-	Partial	Linked Set 41	
ENSG00000204382	ENSG00000204379	0.0000	0.0000	0.414	X/X	8341	+/-	Complete	142156	Linked Set 42
ENSG00000155622	ENSG00000185751	0.0000	0.0000	0.536	X/X	8341	+/-	Complete	Linked Set 42	
ENSG00000183461	ENSG00000204382	0.0000	0.0000	0.504	X/X	179068	+/+	Complete	93872	Linked Set 43
ENSG00000204376	ENSG00000204379	0.0000	0.0000	0.470	X/X	179068	-/-	Complete	Linked Set 43	
