# Supplementary material for: Early evolutionary history and genomic features of gene duplicates in the human genome
Source: BMC Genomics. 2015 Aug 20;16(1):621. doi: 10.1186/s12864-015-1827-3 (PMC4546093; doi:10.1186/s12864-015-1827-3)

Supplementary Figures

Supplementary Figure S1. Decrease in the frequency of *intrachromosomal* duplicates with K_S in three age-cohorts. The results do not markedly change when paralogs on the sex chromosomes are excluded from the analysis.

Supplementary Figure S2. The physical distance between intrachromosomal paralogs as a function of KS. No significant change is observed when paralogs residing on sex chromosomes are included or excluded.

Supplementary Figure S3. Duplication span of DNA- and RNA-mediated duplicates as a function of K_S. A significant decreasing trend for DNA-mediated duplication span is observed irrespective of whether paralogs on sex chromosomes are included or excluded.

Supplementary Figure S4. Transcriptional orientation (*direct* versus *inverse*) of *intrachromosomal* duplicates within three age-cohorts. The proportions of direct and inversely oriented *intrachromosomal* paralogs in not statistically different among the three age cohorts ($K_S = 0$, $0 < K_S \leq 0.025$, and $0.025 < K_S \leq 0.1$). The inclusion or exclusion of paralogs residing on sex chromosomes does not alter the observed pattern.

Supplementary Figure S5. Frequencies of three structural categories of DNA-mediated gene duplicates across three age-cohorts. The relative proportions of *complete*, *partial*, and *chimeric* duplicates shows no significant change with evolutionary time when paralogs residing on sex chromosomes are included or excluded.

Supplementary Figure S6. Box plot displaying the distribution of minimum duplication span for young gene duplicates. The median duplication span is significantly larger than the median coding region length. This pattern is not altered upon the exclusion of paralogs located on (i) the Y chromosome (W = 1461990, $p = 6.38 \times 10^{-5}$) or (ii) on both sex chromosomes (W = 1231118, $p = 1.05 \times 10^{-5}$).

Supplementary Figure S1

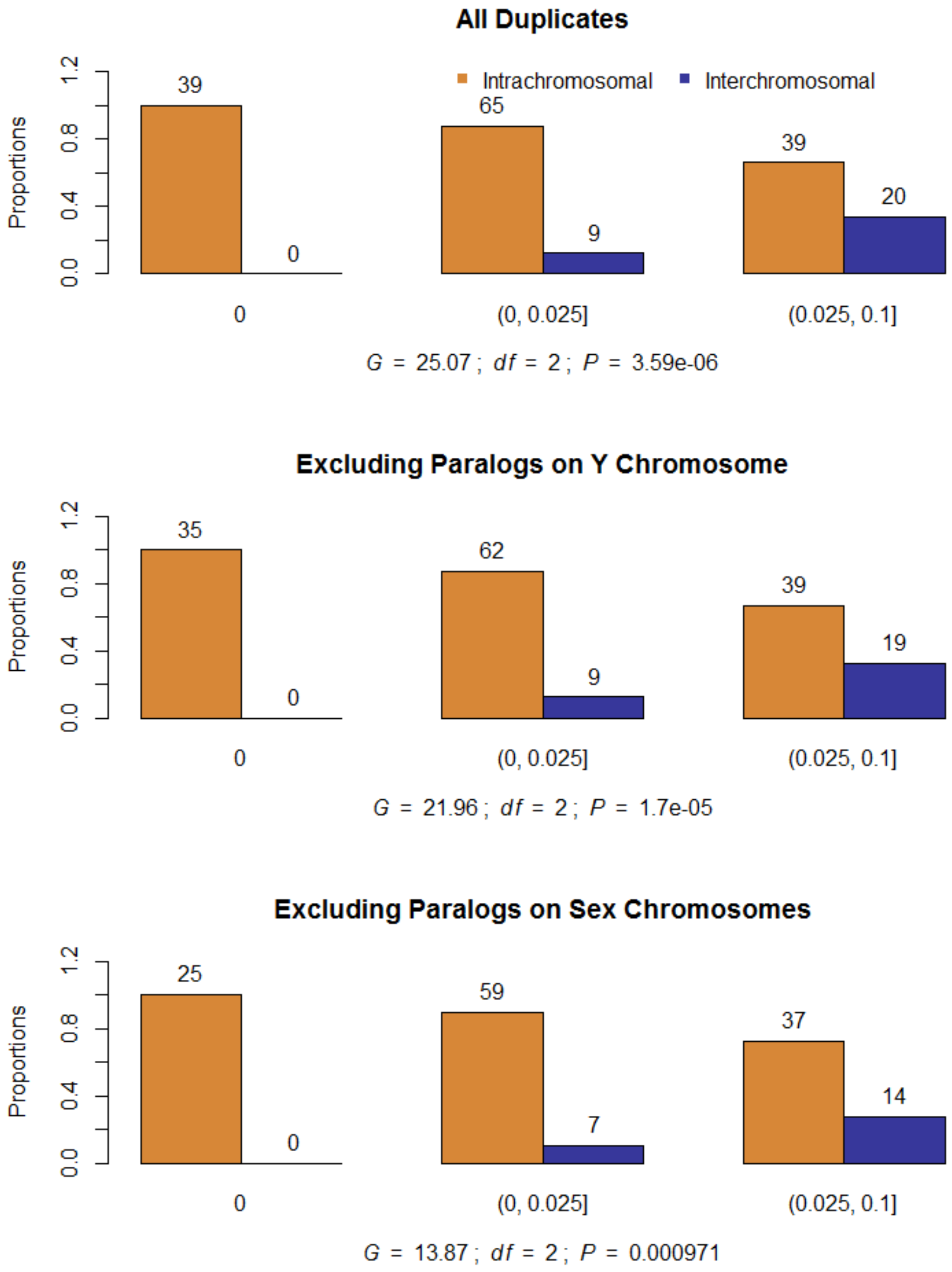

Supplementary Figure S2

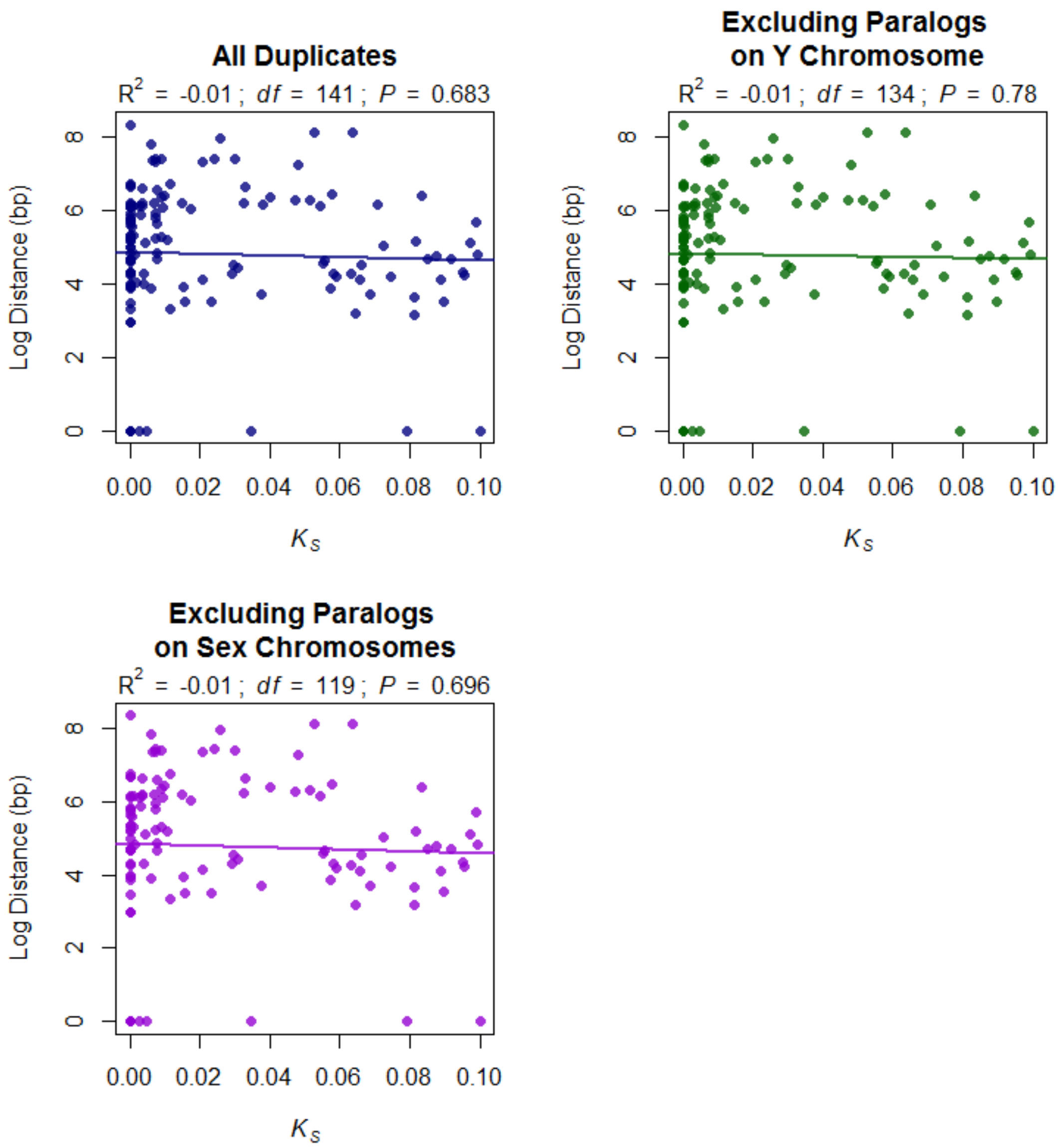

Supplementary Figure S3

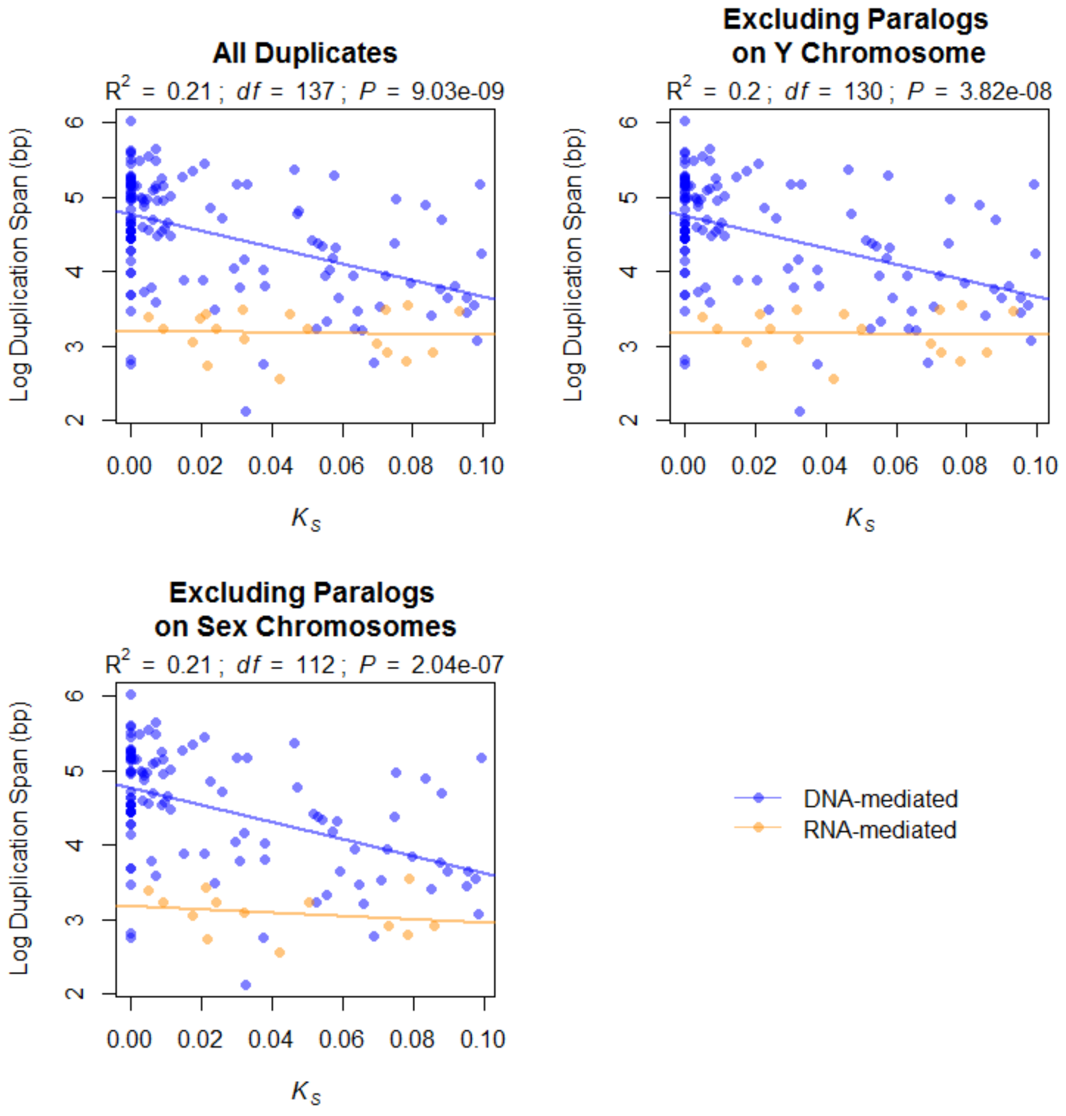

Supplementary Figure S4

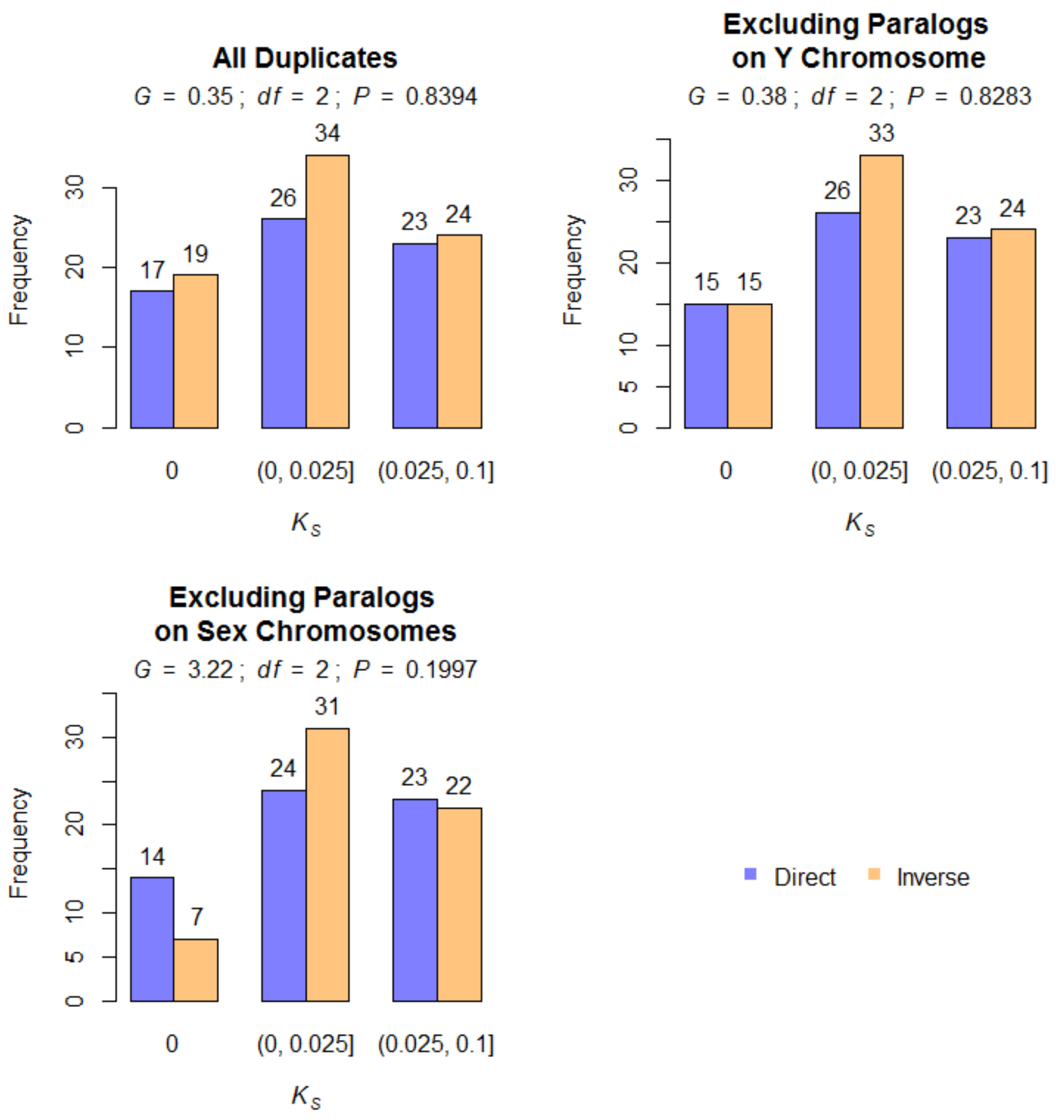

Supplementary Figure S5

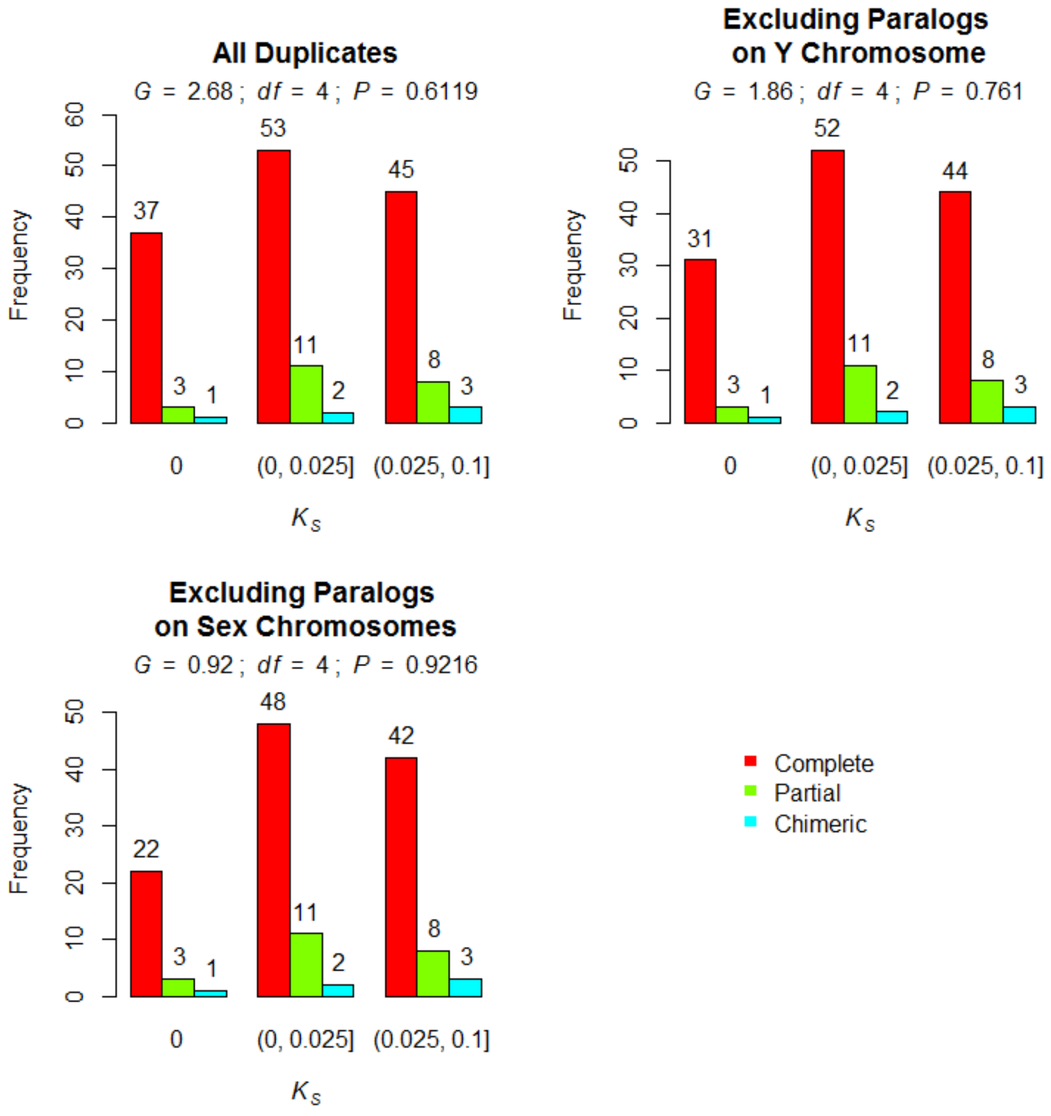

Supplementary Figure S6

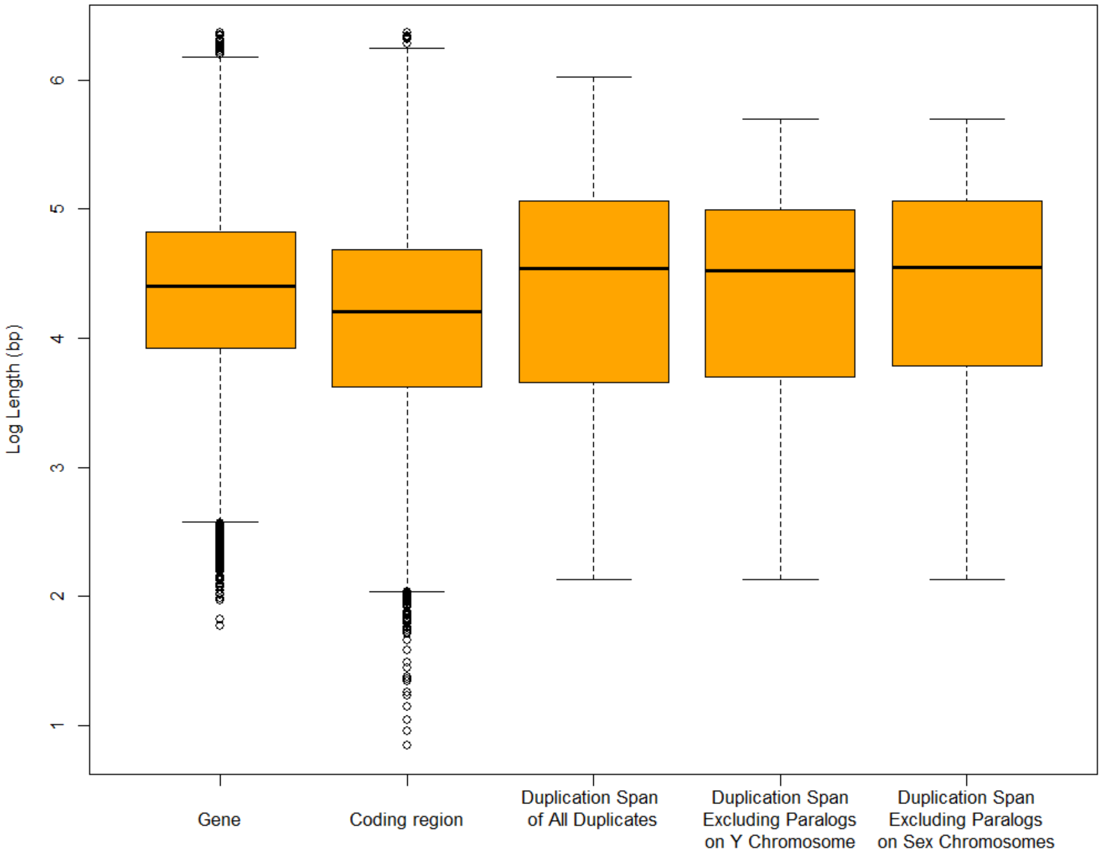

Supplement: Additional file 2: Figure S1. — Decrease in the frequency of intrachromosomal duplicates with K S in three age-cohorts. Figure S2. The physical distance between intrachromosomal paralogs as a function of K S. Figure S3. Duplication span of DNA- and RNA-mediated duplicates as a function of K S. Figure S4. Transcriptional orientation (direct versus inverse) of intrachromosomal duplicates within three age-cohorts. Figure S5. Frequencies of three structural categories of DNA-mediated gene duplicates across three age-cohorts. Figure S6. Box plot displaying the distribution of minimum duplication span for young gene duplicates. (PDF 303 kb) [file 12864_2015_1827_MOESM2_ESM.pdf]
